# The Effectiveness of National Expanded Program on Immunization With Hepatitis A Vaccines in the Chinese Mainland: Interrupted Time-Series Analysis

**DOI:** 10.2196/53982

**Published:** 2024-02-28

**Authors:** Bo-Wen Ming, Li Li, Hao-Neng Huang, Jia-Jun Ma, Chen Shi, Xiao-Han Xu, Zhou Yang, Chun-Quan Ou

**Affiliations:** 1 State Key Laboratory of Organ Failure Research, Department of Biostatistics School of Public Health Southern Medical University Guangzhou China

**Keywords:** hepatitis A, incidence, Expanded Program on Immunization, vaccine, interrupted time series, intervention, China

## Abstract

**Background:**

The high prevalence of hepatitis A delivered a blow to public health decades ago. The World Health Organization (WHO) set a goal to eliminate viral hepatitis including hepatitis A by 2030. In 2008, hepatitis A vaccines were integrated into the Expanded Program on Immunization (EPI) in China to alleviate the burden of hepatitis A, although the effectiveness of the EPI has not been well investigated.

**Objective:**

We aimed to evaluate the intervention effect at both provincial and national levels on the incidence of hepatitis A in the Chinese mainland from 2005 to 2019.

**Methods:**

Based on the monthly reported number of hepatitis A cases from 2005 to 2019 in each provincial-level administrative division, we adopted generalized additive models with an interrupted time-series design to estimate province-specific effects of the EPI on the incidence of hepatitis A among the target population (children aged 2-9 years) from 2005 to 2019. We then pooled province-specific effect estimates using random-effects meta-analyses. We also assessed the effect among the nontarget population and the whole population.

**Results:**

A total of 98,275 hepatitis A cases among children aged 2-9 years were reported in the Chinese mainland from 2005 to 2019, with an average annual incidence of 5.33 cases per 100,000 persons. Nationally, the EPI decreased the hepatitis A incidence by 80.77% (excess risk [ER] –80.77%, 95% CI –85.86% to –72.92%) during the study period, guarding an annual average of 28.52 (95% empirical CI [eCI] 27.37-29.00) cases per 100,000 persons among the target children against hepatitis A. Western China saw a more significant effect of the EPI on the decrease in the incidence of hepatitis A among the target children. A greater number of target children were protected from onset in Northwest and Southwest China, with an excess incidence rate of –129.72 (95% eCI –135.67 to –117.86) and –66.61 (95% eCI –67.63 to –64.22) cases per 100,000 persons on average, respectively. Intervention effects among nontarget (ER –32.88%, 95% CI –39.76% to –25.21%) and whole populations (ER –31.97%, 95% CI –39.61% to –23.37%) were relatively small.

**Conclusions:**

The EPI has presented a lasting positive effect on the containment of hepatitis A in the target population in China. The EPI’s effect on the target children also provided a degree of indirect protection for unvaccinated individuals. The continuous surveillance of hepatitis A and the maintenance of mass vaccination should shore up the accomplishment in the decline of hepatitis A incidence to ultimately achieve the goal set by the WHO.

## Introduction

Hepatitis A is an acute infectious disease that is transmitted via a fecal-oral route, primarily through the ingestion of food or water contaminated with the virus [1-3]. Hepatitis A would frequently give rise to sporadic cases, outbreaks, and even epidemics [4,5], which posed threats to public health and laid an excessive economic burden on society. Globally, the burden of hepatitis A has been increasing in recent years [6], accounting for about 1.4 disability-adjusted life years (DALYs) and 2.3 DALYs in 2010 and 2019, respectively [7,8]. Given the current global burden of hepatitis A viruses (HAV), the World Health Organization (WHO) developed a long-term goal of eliminating viral hepatitis, including hepatitis A, as a major public health threat by 2030 [9].

Vaccination is an essential intervention for the prevention and control of hepatitis A. Assessing the effectiveness of vaccination on the incidence of hepatitis A is a critical step to the continuous implementation and improvement of the immunization program. Although some studies have investigated the correlation between vaccination coverage and hepatitis A incidence [10] or seroprevalence [11,12], there are limited data on the intervention effect of hepatitis A mass vaccination. A few studies simply described and compared the average yearly incidence between the pre- and postintervention periods in the Chinese mainland and Israel [4,13-17], which neglected potential confounding factors. Two studies used regression models to evaluate the association of the vaccination program with the incidence of hepatitis A in the Chinese mainland and Brazil [17,18]. However, these studies did not consider the potential regional differences in the effect of the vaccination, which would fail to promote precise and differentiated strategies in the prevention and control of hepatitis A. Another research study assessed the impact of the Expanded Program on Immunization (EPI) on the incidence of 11 childhood vaccine-preventable diseases, including hepatitis A, with annual data in the Chinese mainland through a quasi-Poisson regression and a polynomial regression [19]. However, this study constructed the model based on national data of the whole population and assumed that all changes in the hepatitis A incidence from 2003 to 2012 were fully attributable to vaccination, thus failing to assess the impact of the EPI in different age groups. Furthermore, the potential spatial and time-varying effects of the EPI on hepatitis A remained unclear.

In the Chinese mainland, hepatitis A was endemic in most regions from 1990 to 2007, with an average annual incidence of 21.31 cases per 100,000 persons, in the absence of systematic hepatitis A inoculation [20]. Furthermore, children under the age of 10 years were subjected to the highest incidence among all age groups from 1990 to 2007, and public health emergencies of hepatitis A occurred mostly in elementary schools in Western China [2,20]. The Chinese government integrated hepatitis A vaccines into the Chinese National EPI in 2008 to mitigate hepatitis A. Children aged over 18 months are eligible to either (1) receive 1 dose of the live attenuated hepatitis A vaccine or (2) receive 1 dose of the inactivated vaccine at the 18th month and another dose at the 24th month for free in the Chinese mainland [10]. However, the initiation time and the intensity of the EPI were different in each provincial-level administrative division (PLAD). Hence, it is necessary to explore the effect of the EPI in different PLADs and then pool the effect estimates to obtain substantial evidence. Herein, we aimed to assess the effectiveness of the EPI in China at the provincial and national levels.

## Methods

### Data Collection

We collected monthly data on hepatitis A incidence across 31 PLADs from 2005 to 2019 in the Chinese mainland from the National Population and Health Science Data Sharing Platform of the Chinese Center for Disease Control and Prevention [21]. A hepatitis A case is defined as a patient with acute gastrointestinal symptoms (eg, jaundice) and elevated serum alanine aminotransferase who tests positive for the immunoglobulin M antibody to HAV [22].

The provincial-level annual data (ie, the age-specific resident population, gross domestic product [GDP] per capita, urbanization rates, the number of hospitalization beds per 1000 persons, the proportion of children younger than 14 years old, and illiteracy rates) from 2005 to 2019 were compiled from China Statistical Yearbooks [23,24]. The monthly resident population was derived from linear interpolation. Monthly data on ambient average temperature were downloaded from the China Meteorological Data Sharing Service System [25].

### Data Analysis

The exact implementation time of the EPI and the use of the vaccine type (ie, inactivated hepatitis A vaccines or live attenuated hepatitis A vaccines) were different among PLADs. Accordingly, the postintervention period of each PLAD was specified separately (Table S1 in Multimedia Appendix 1). Children born after 2007 and aged >24 months are eligible to receive hepatitis A vaccines. The postintervention period spanned 11 years. Children younger than 13 years old could directly benefit from the program at the end of the study period. However, the raw data were in the form of 1-year age groupings for those aged <10 years and 5-year age groupings after the age of 10 years (10-14 years, 15-19 years, etc). Hence, we identified children aged 2-9 years whom the EPI had a direct bearing on as the study population.

A 2-stage analytic strategy was used to evaluate the effect of the EPI on the incidence of hepatitis A. In the first stage, a quasi-Poisson regression model with interrupted time series was established to capture the province-specific effectiveness of the EPI after adjusting for covariates in each of the 30 PLADs [26]. Tianjin was not considered because the average annual number of cases was extremely low even before the EPI (3.55 cases per year; Table S2 in Multimedia Appendix 1), which cannot ensure the stability of modeling. The model mainly contains an elapsed time since the start of the study, a dummy variable indicating the pre- or postintervention period, and an interaction term for the aforementioned 2 variables. Furthermore, we included the natural cubic spline of monthly average temperature with 3 *df*s, which was selected by minimizing the value of quasi–Akaike information criterion (Table S3 in Multimedia Appendix 1) and a categorical variable of calendar months to control for the potential seasonality of hepatitis A incidence. We incorporated the reported public health emergencies of hepatitis A, mainly occurring before the implementation of the EPI, into the model to avoid the overestimation of the effects (Table S4 in Multimedia Appendix 1) [27]. An autoregressive term of residuals at lag 1 was taken into account to adjust for autocorrelation if necessary (Table S5 and Figures S1 and S2 in Multimedia Appendix 1). Detailed information on the provincial-specific model is provided in the Supplemental Methods in Multimedia Appendix 1.

The provincial-specific excess risk (ER) of the hepatitis A incidence and the corresponding 95% CI were estimated. In addition, the excess incidence rate (EIR) in each province and the corresponding 95% empirical CI (eCI) were estimated to investigate the change in the hepatitis A incidence due to the implementation of the EPI. The details on the estimation of ERs and EIRs are demonstrated in the Supplemental Methods in Multimedia Appendix 1.

In the second stage, we pooled the province-specific effect of the EPI using random-effects meta-analyses to derive the regional-level and national-level estimates of the ER. The heterogeneity was quantified by *I*^2^ statistic and Cochran Q test [28]. In addition, we estimated the national population-weighted EIRs.

Furthermore, we performed subgroup analyses. The PLADs were classified into 7 regions (ie, Northeast, North, Northwest, East, Center, South, and Southwest), considering different geographical and environmental conditions [29]. We also estimated the EIR for each subgroup of PLADS by socioeconomic factors (ie, urbanization rates, GDP per capita, the number of hospitalization beds per 1000 persons, the proportion of children aged 0-14 years, and illiteracy rates), the level of average incidence of hepatitis A before the implementation of EPI, and the types of hepatitis A vaccines (Table S1 in Multimedia Appendix 1). All factors, except for regions and types of hepatitis A vaccines, were classified into 2 levels using the medians as the cutoffs (high: ≥median; low: <median).

In addition, we explored the effect of the EPI on the whole population and the nontarget population (children younger than the age of 2 years or older than the age of 10 years), as previous literature suggested that the vaccination might develop herd immunity against hepatitis A [10]. We did not consider the interaction term between time and the indicator variable of the intervention in models for the nontarget population and the whole population because the pooling effects of the interaction term fell short of statistical significance.

We performed sensitivity analyses to examine the robustness of the main model results (Supplemental Methods in Multimedia Appendix 1). First, we substituted calendar months with a natural cubic spline function with 3 *df*s to consider different ways of controlling potential seasonality. Second, we explored the potentially nonlinear intervention effect over time. Finally, we took the transition period from the implementation of the EPI to 2010 into account and excluded this period from the analyses. A 2-sided *P* value of <.05 was considered statistically significant. We used the R statistical software (version 4.2.1; R Foundation for Statistical Computing) to perform all analyses.

### Ethical Consideration

This study was approved by the Research Ethics Committee of Southern Medical University (NFYKDX-ER2022012). The need for informed consent was waived because the data were deidentified and aggregated.

## Results

### Temporal and Spatial Distribution of Hepatitis A Incidence

Table 1 presents the summary statistics of hepatitis A in different age groups from 2005 to 2019 in the Chinese mainland. During the study period, there was a total of 98,275 reported hepatitis A cases among children aged 2-9 years with an average annual incidence of 5.33 cases per 100,000 persons. Overall, there was a gradual decline in the annual incidence of hepatitis A. The incidence of hepatitis A increased from 10.98 cases per 100,000 persons in 2005 to 16.80 cases per 100,000 persons in 2007, but the incidence had considerably dropped since 2008 and was only 0.56 cases per 100,000 persons in 2019. Among the whole population, 556,737 cases were reported from 2005 to 2019, with an average annual 2.79 cases per 100,000 persons. The incidence among the whole population and the nontarget population also appeared to be edging downward, with the latter leveling off at 1.50 cases per 100,000 persons after 2011.

**Table 1:** The annual number of cases and hepatitis A incidence in the Chinese mainland.

Year	Target population^a^			Nontarget population			Whole population	
	Cases, n	Incidence (cases per 100,000 persons)	Cases, n		Incidence (cases per 100,000 persons)	Cases, n		Incidence (cases per 100,000 persons)
2005^b^	13,375	10.98	59,966		5.09	73,349		5.64
2006	13,602	11.29	55,065		4.64	68,667		5.25
2007	20,325	16.80	56,810		4.76	77,135		5.87
2008	11,186	9.15	44,866		3.74	56,052		4.24
2009	7958	6.41	35,883		2.98	43,841		3.30
2010	7317	5.66	27,960		2.32	35,277		2.64
2011	4969	4.22	26,487		2.17	31,456		2.35
2012	3598	2.95	20,855		1.70	24,453		1.81
2013	2852	2.33	19,392		1.57	22,244		1.64
2014	4604	3.68	21,365		1.72	25,969		1.92
2015	2763	2.17	19,904		1.60	22,667		1.66
2016	2449	1.91	18,836		1.52	21,285		1.55
2017	1554	1.20	17,321		1.39	18,875		1.37
2018	1017	0.77	15,179		1.21	16,196		1.17
2019	706	0.56	18,565		1.46	19,271		1.38
Total	98,275	5.33	458,454		2.52	556,737		2.79

^a^The target population is children aged 2-9 years.

^b^There were 8 cases whose ages were missing.

**Figure 1:**
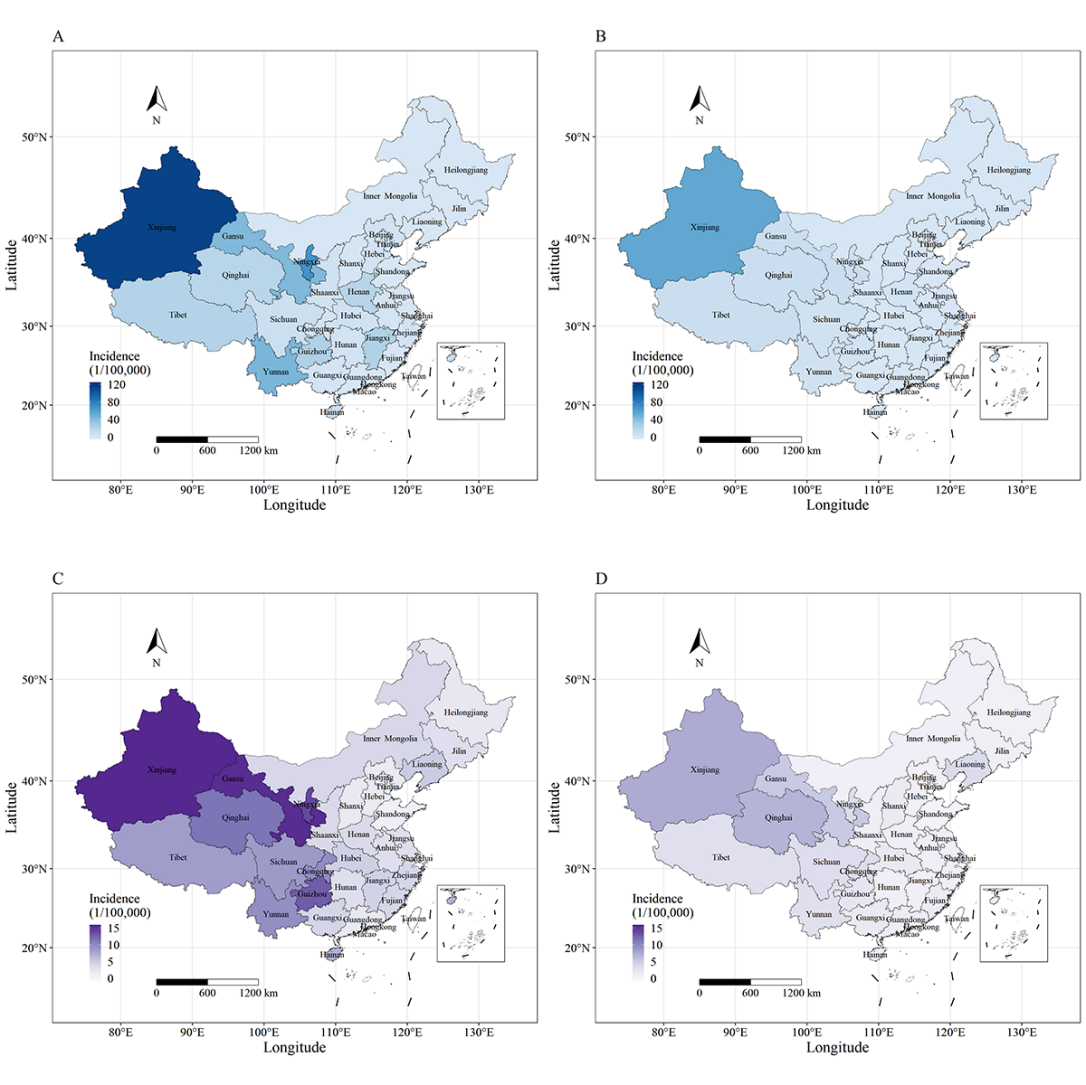
Spatial distribution of the average annual hepatitis A incidence. White regions indicate missing data. (A) Preintervention period among children aged 2-9 years; (B) postintervention period among children aged 2-9 years; (C) preintervention period among nontarget individuals; (D) postintervention period among nontarget individuals.

Figure 1 demonstrates the spatial distribution of the average hepatitis A incidence among children aged 2-9 years and the nontarget population. Generally, Western China witnessed a higher incidence of hepatitis A than that of other regions during both the pre- and postintervention periods. However, this regional difference has remarkably dwindled since the implementation of the EPI (Table S6 in Multimedia Appendix 1). The hepatitis A incidence among children aged 2-9 years showed significant seasonal variation, which peaked in the autumn and winter. However, the seasonality was gradually ambiguous, and the peak slumped greatly in the postintervention stage (Figures S3 and S4 in Multimedia Appendix 1). The seasonality pattern of Western China was similar, but the seasonality was not apparent in other regions.

### Effect of EPI on Hepatitis A Incidence

**Figure 2:**
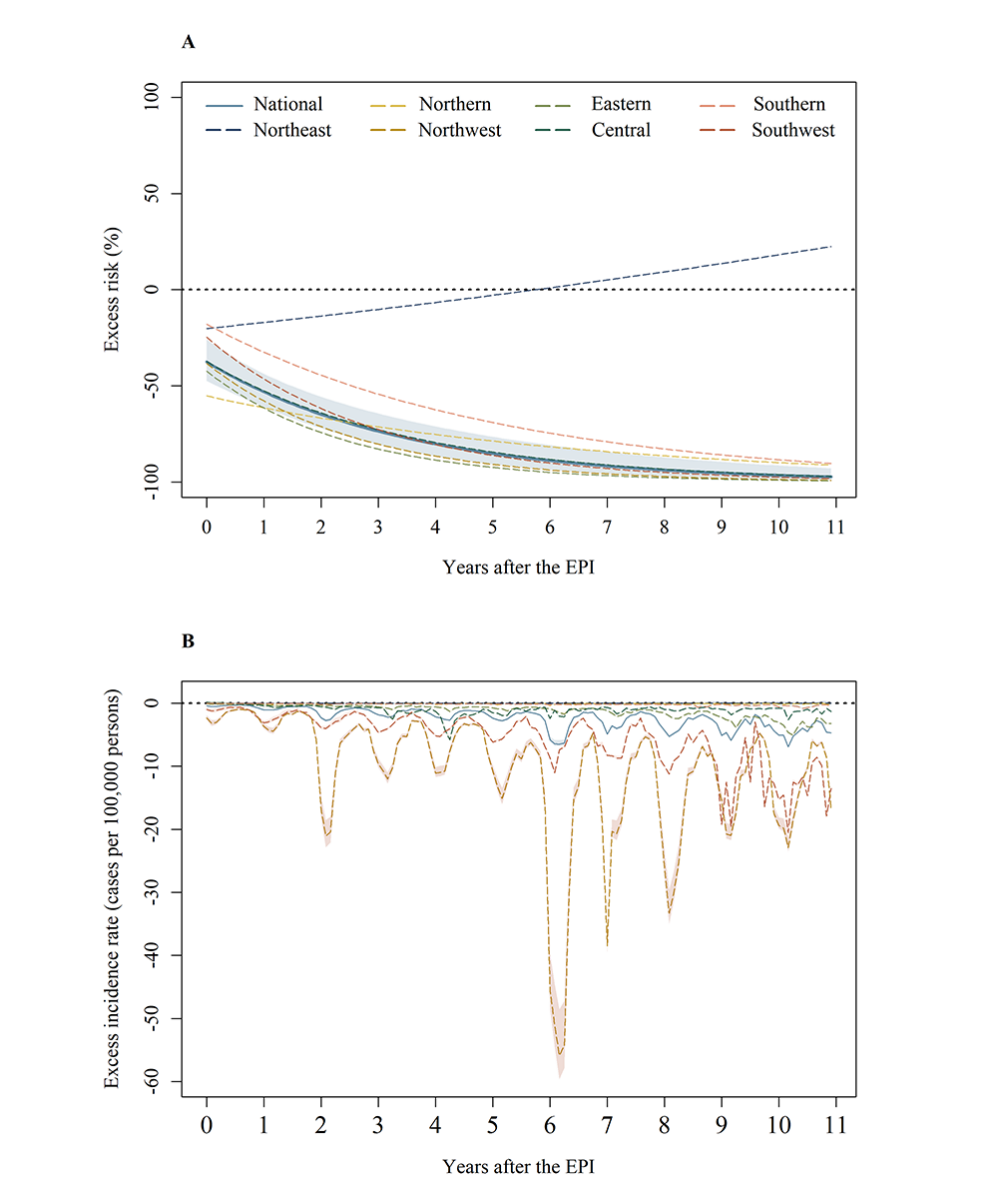
Excess risks and excess incidence rates towards children aged 2-9 years. The line represents the point estimates of excess risks and excess incidence rates of hepatitis A associated with the EPI. The colored regions are the corresponding 95% CIs and 95% empirical CIs, respectively. The excess risks of Northeast China are not statistical significant during the overall period after the implementation of the EPI. (A) Excess risks; (B) excess incidence rates. EPI: Expanded Program on Immunization.

Overall, the implementation of the EPI led to a significant decrease in the incidence of hepatitis A for the target population, with an average ER of 80.77% (95% CI 72.92%-85.86%) among children aged 2-9 years (Figure 2 and Figure S5 in Multimedia Appendix 1). The magnitude of the EPI intervention enhanced with each passing year from the initiation of the EPI. Specifically, the intervention impact was relatively weak in the first year after the implementation of the EPI, associated with a 51.92% (95% CI 42.44%-59.84%) reduction in the incidence of hepatitis A. The reduction dramatically rose to 97.25% (95% CI 92.77%-98.95%) in the 11th year following the EPI (Table S7 in Multimedia Appendix 1).

Table 2 and Tables S8 and S9 in Multimedia Appendix 1 show the EPI-associated reduction in cases and the incidence of hepatitis A among children aged 2-9 years. In general, it was estimated that a yearly average of 28.52 (95% eCI 27.37-29.00) hepatitis A cases per 100,000 persons was averted within 11 years of intervention. In other words, on average, about 34,618 cases are prevented each year by the EPI. In detail, the EPI protected an average of 14.31 (95% eCI 13.50-14.78) cases per 100,000 persons from hepatitis A in the first 5 years of the intervention period. In the subsequent 6-11 years, on average, 39.90 (95% eCI 38.34-40.48) cases were prevented per 100,000 individuals.

**Table 2:** Annual average excess number of cases and excess incidence rate of hepatitis A in target children.

Variable and region			1-5 years after the EPI^a^		6-11 years after the EPI		1-11 years after the EPI
**Excess number of case (95% empirical CI; 1000 cases)**							
	Chinese mainland	–16.99 (–17.55 to –16.03)		–49.31 (–50.03 to –47.38)		–34.62 (–35.20 to –33.22)	
	Northeast	–0.01 (–0.01 to 0.03)		–0.01 (–0.01 to 0.10)		–0.01 (–0.01 to 0.07)	
	North	–0.20 (–0.22 to –0.16)		–0.23 (–0.24 to –0.15)		–0.21 (–0.23 to –0.16)	
	Northwest	–6.34 (–6.72 to –5.77)		–18.51 (–19.31 to –16.79)		–12.98 (–13.57 to –11.79)	
	East	–1.76 (–1.84 to –1.65)		–7.77 (–7.79 to –7.69)		–5.04 (–5.08 to –4.95)	
	Center	–2.35 (–2.62 to –1.96)		–2.95 (–2.99 to –2.88)		–2.68 (–2.82 to –2.46)	
	South	–0.20 (–0.24 to –0.13)		–0.57 (–0.60 to –0.48)		–0.40 (–0.43 to –0.32)	
	Southwest	–6.14 (–6.46 to –5.55)		–19.27 (–19.41 to –18.79)		–13.30 (–13.50 to –12.82)	
**Excess incidence rate (95% empirical CI; cases per 100,000 persons)**							
	Chinese mainland	–14.31 (–14.78 to –13.50)		–39.90 (–40.48 to –38.34)		–28.52 (–29.00 to –27.37)	
	Northeast	–0.07 (–0.20 to 0.37)		–0.08 (–0.19 to 1.49)		–0.07 (–0.19 to 0.96)	
	North	–1.47 (–1.64 to –1.18)		–1.58 (–1.67 to –1.06)		–1.53 (–1.65 to –1.14)	
	Northwest	–63.25 (–67.06 to –57.55)		–185.28 (–193.32 to –168.03)		–129.72 (–135.67 to –117.86)	
	East	–5.50 (–5.72 to –5.14)		–22.37 (–22.42 to –22.12)		–15.04 (–15.15 to –14.77)	
	Center	–11.95 (–13.32 to –9.96)		–13.35 (–13.50 to –13.00)		–12.75 (–13.42 to –11.71)	
	South	–1.23 (–1.47 to –0.78)		–3.53 (–3.68 to –2.92)		–2.49 (–2.68 to –1.97)	
	Southwest	–30.01 (–31.61 to –27.12)		–98.50 (–99.25 to –96.05)		–66.61 (–67.63 to –64.22)	

^a^EPI: Expanded Program on Immunization.

There were significant differences among provinces in the effect of the EPI intervention on the incidence of hepatitis A among children aged 2-9 years (*I*^2^=92.80%; *P*<.001). We observed significant effects in Northwest (ER –99.02%, 95% CI –99.69% to –96.87%) and Eastern (ER –99.29%, 95% CI –99.90% to –95.12%) China during the whole intervention period (Figure 2 and Table S10 in Multimedia Appendix 1), while the ER in Northeast China was statistically nonsignificant. Table 2 and Tables S8 and S9 in Multimedia Appendix 1 reflect the moderate effectiveness of the intervention on the target children in Northern, Central, and Southern China. Compared with other regions, a greater number of the target children were protected in Northwest and Southwest China, with an EIR of –129.72 (95% eCI –135.67 to –117.86) and –66.61 (95% eCI –67.63 to –64.22) cases per 100,000 persons on average, respectively.

**Figure 3:**
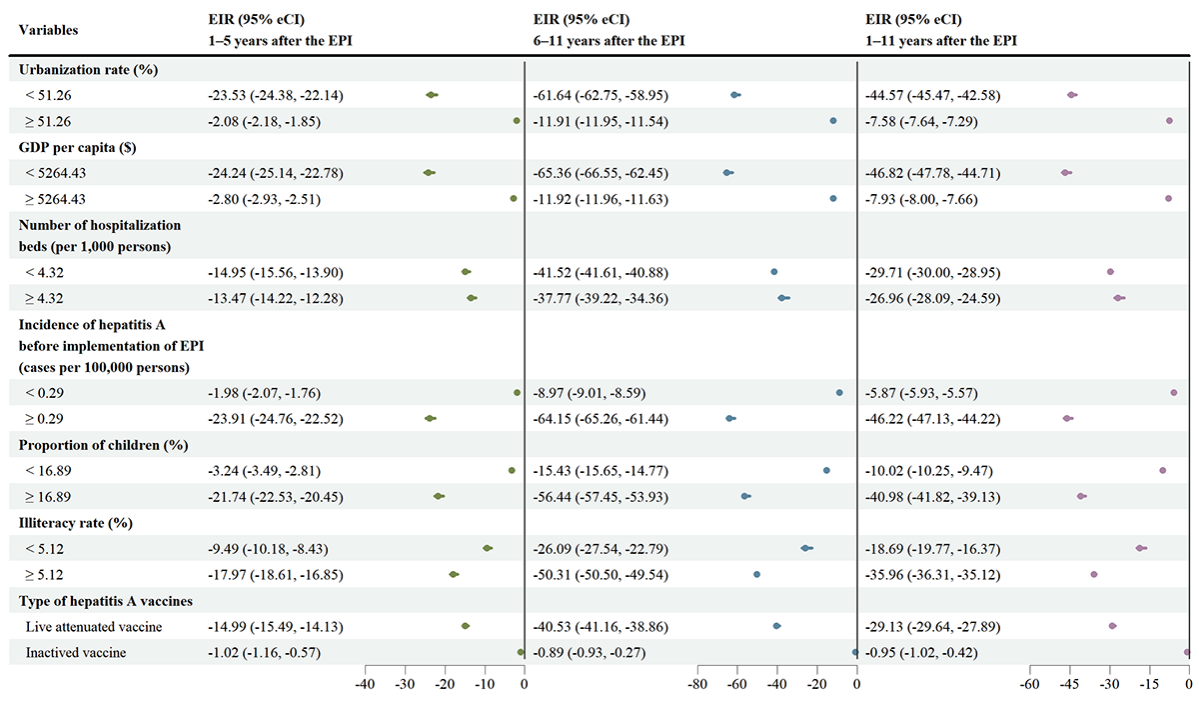
Average excess incidence rates in different subgroups among children aged 2-9 years. eCI: empirical CI; EIR: excess incidence rate.

Figure 3 and Table S9 and Figure S6 in Multimedia Appendix 1 provide ERs and EIRs of the EPI for different subgroups based on socioeconomic factors and types of hepatitis A vaccines. The ERs of hepatitis A incidence in the intervention population did not differ greatly at different socioeconomic levels and vaccine types. However, EIRs exhibited greater differences between subgroups than ERs. PLADs with less GDP per capita, lower urbanization rates, insufficient number of hospitalization beds per 1000 persons, higher incidence of hepatitis A before the implementation of the EPI, larger proportion of children younger than 14 years old, higher illiteracy rates, or live attenuated hepatitis A vaccines witnessed a more significant alleviation in the incidence of hepatitis A during the whole intervention period.

In view of all people and nontarget people, the EPI also lessened the burden of hepatitis A (Tables S11 and S12 in Multimedia Appendix 1). Generally, the EPI was likewise linked to the decrease in the incidence in nontarget individuals (ER –32.88%, 95% CI –39.76% to –25.21%) and all people (ER –31.97%, 95% CI –39.61% to –23.37%). The EPI could prevent 0.78 (95% eCI 0.73-0.82) and 0.83 (95% eCI 0.78-0.88) cases per 100,000 persons among nontarget individuals and all people from infection within 11 years of the intervention, respectively.

### Sensitivity Analyses

We obtained similar results from the sensitivity analyses (Table S7 and Figures S7-9 in Multimedia Appendix 1). First, the effect of the EPI on the incidence did not change substantially when using a natural cubic spline to control for seasonality (ER –80.25%, 95% CI –85.66% to –71.68%). Second, in accordance with the main results, the exposure-response curves revealed that the logarithm transformation of ER in large changed linearly with time. Finally, the results, in consideration of the transition period, were similar to those in the main analysis, with an average ER of 80.45% (95% CI 71.19%-86.48%).

## Discussion

### Principal Findings

In this nationwide study, we comprehensively assessed the effectiveness of the EPI on the incidence of hepatitis A among children aged 2-9 years based on province-specific monthly data from 2005 to 2019. The 97% decrease in hepatitis A incidence attributable to the 11-year intervention of the EPI among children aged 2-9 years provides an estimate of the vaccine-preventable HAV cases in childhood, suggesting that the burden of the hepatitis A cyclical epidemic before the EPI has been largely alleviated [10]. By the end of 2019, the incidence of hepatitis A decreased to 0.56 cases per 100,000 persons among target children in the Chinese mainland, which gradually approaches the target of eliminating hepatitis A set by the WHO.

Two previous studies have shown the beneficial effectiveness of the EPI in curbing the incidence of hepatitis A in the Chinese mainland [10,17]. Sun et al [10] described an 82.3% decline in the incidence in 2016 compared to that in 2004. Tang et al [17] indicated that 47% of the decrease in the incidence of hepatitis A among children aged 0-14 years was attributable to the EPI, by simply fitting the annual number of cases using an interrupted linear regression without considering the nonlinear trend and the change in the population size, which might bring about bias in the estimates of the intervention effect. A previous review in China demonstrated that seroconversion rates were well above 80% and vaccine efficacy rates were higher than 90% in vaccinated individuals, whether with live attenuated hepatitis A vaccines or inactivated hepatitis A vaccines [5]. A randomized clinical trial indicated that nearly 100% of vaccinated people developed serum antibodies against HAV after the inoculation [30]. In this population-based interrupted time-series study, we used a robust 2-stage modeling strategy to comprehensively assess the province-specific effect and the pooled national effect. Combined with the previous evidence, this study could corroborate that the EPI did weigh positively on the decrease of hepatitis A incidence among the vaccinated population.

In addition, there were distinct regional differences in the effect of the EPI, with the greatest effect in Western China. The effects on the target children were not statistically significant in several PLADs of Eastern and Northern China where the incidence was low prior to the intervention. A possible explanation may be that a portion of schoolchildren had received self-financed vaccination against hepatitis A before the implementation of the EPI due to better economic conditions and awareness of vaccination in these PLADs compared to Western China [10,31]. The annual number of hepatitis A cases in Tianjin fluctuated between 0 and 6 cases among the target children during the study period from 2005 to 2019. One possible reason was that the local immunization strategy was implemented in 2001, covering 1-year-old infants, first-grade children, and seventh-grade children [31,32]. The hepatitis A vaccine coverage was above 90% in 2001, which facilitated a low-level sporadic state of hepatitis A in Tianjin among children [31,32]. Notably, the postintervention incidence of hepatitis A in Western China, especially in Tibet and Xinjiang, was still significantly higher than that of other regions of China, although the EPI had largely alleviated the local burden of hepatitis A. These discrepancies might be related to impoverishment [24] and the lack of vaccination awareness. For example, the vaccination coverage in children was only about 60% in Tibet in 2004, and about 71% of towns in Xinjiang achieved the goal of 90% coverage of hepatitis A vaccine in 2013 [10,33,34]. A sampling survey found a lower anti-HAV antibody seroprevalence in Western China than in Eastern China (69% vs 72% in 2014) [11]. None of the local health authorities could afford to overlook the gaps in the vaccination coverage among children between the Western region (69.1%) and other regions of China (86% in Eastern China and 74.5% in Middle China) [33]. Further promotion of vaccination and prevention measures against hepatitis A in Western China, including raising public awareness about the prevention of hepatitis A and the efficacy of vaccination, remains a long way off to narrow the gaps. However, there is no denying that the massive disparities in the geographical distribution of the hepatitis A incidence before the intervention, with the vast majority of cases in the Western region, have faded away in most PLADs as the intervention progressed. Overall, focusing on the reduction of HAV exposure and health education on vaccination against hepatitis A among local children in high-prevalence regions will enhance the effectiveness of the intervention and thereby lighten the burden of hepatitis A.

Subgroup analyses indicated that there is no significant difference in reducing the relative risk of hepatitis A onset between regions with different socioeconomic conditions and types of hepatitis A vaccines. However, it is worth noting that the decrease in the incidence of hepatitis A due to the implementation of the EPI varied evidently across PLADs with different socioeconomic levels and types of vaccines. Specifically, the alleviation of the hepatitis A burden in resource-limited regions was much higher than that in resource-rich regions, which are mainly concentrated in Eastern China. Similarly, greater effectiveness was found in PALDs with live attenuated vaccines (ie, all PLADs except Beijing, Tianjin, Shanghai, and Jiangsu). Differences in EIRs could be boiled down to 2 reasons, namely, the level of development and the incidence before the implementation of the EPI. The regions where society and economy have forged ahead during the last decade (ie, high GDP per capita, the fast urbanization process, sufficient medical resources, decreased proportion of children, or advanced education level), accompanied by improvements in sanitary and hygienic conditions (ie, sufficient medical resources), are associated with the less exposure to HAV, which could partly explain the regional difference in EIRs [24,35]. More could be credited to the fact that there were lower incidence of hepatitis A and higher seroprevalence of anti-HAV antibody in regions with thriving economy before the intervention [11].

In terms of the nonintervention population beyond the age group of 2-9 years, the EPI-associated reduction in the incidence of hepatitis A was observed. This finding indicated that routine mass immunization on target children provided a degree of indirect protection for unvaccinated individuals, in line with a previous study in the United States [36]. With fewer hepatitis A cases in target children, the probability of cross-infection from target children to nontarget people reduces greatly. On the other hand, among nonvaccinated individuals, people who were neither covered by the EPI nor had paid for hepatitis A vaccines out of pocket remain vulnerable to hepatitis A. Herd immunity can prevent the spread of hepatitis A in the population to a certain degree but will not adequately protect susceptible individuals upon exposure to HAV [36,37]. Hence, health authorities need to provide education on scientific prevention directives and formulate supervisory policies in light of different infection sources and transmission routes, such as monitoring HAV circulation [38], safeguarding food safety [39], and raising health consciousness, to pave the way for nontarget people to avoid HAV exposure.

### Limitations

Several limitations should be acknowledged in this study. First, all hepatitis A cases were diagnosed and reported by hospitals. There is inevitable underreporting. Particularly, children are more likely to become asymptomatic and be underreported. The underreporting decreases with the improvement of the diagnosis process, which could lead to the underestimation of the effectiveness. However, the odds favor a random bias on asymptomatic cases before and after the implementation of the EPI, thus exerting nondifferential impacts on the assessment of the intervention. Second, the lack of the data on hepatitis A vaccination coverage and the seroprevalence blocks us from better understanding the pathway and the regional heterogeneity of the EPI’s effects on the hepatitis A incidence. Finally, our study emphasized the effect of the EPI on the incidence. The economic benefits and decreased DALYs brought by the EPI have not been investigated. Further dynamic research is greatly needed to articulate the substantial positive effect on controlling the HAV endemic in terms of cost-effectiveness after decades of the EPI and to further evaluate the feasibility of achieving WHO 2030 targets.

### Conclusion

The implementation of EPI has evidently contributed to the decrease of hepatitis A incidence among children aged 2-9 years in the Chinese mainland, suggesting that continuous children mass vaccination is imperative in the future containment of hepatitis A. In view of the regional differences in the effectiveness of the EPI observed, sustained monitoring of the incidence and strengthening of vaccination in Western China will accelerate efforts toward achieving the target of eliminating viral hepatitis set by the WHO. Furthermore, the findings would serve as a favorable paradigm of mass hepatitis A vaccination for other countries and regions with a heavy burden of hepatitis A, which contributes to the eventual elimination of hepatitis A worldwide.

## Appendix

Multimedia Appendix 1 Supplemental methods, tables, and figures.

## Abbreviations

DALY: disability-adjusted life year
eCI: empirical confidence interval
EIR: excess incidence rate
EPI: Expanded Program on Immunization
ER: excess risk
GDP: gross domestic product
HAV: hepatitis A viruses
PLAD: provincial-level administrative division
WHO: World Health Organization

## Data Availability

Relevant raw data related to reported cases of hepatitis A in the Chinese mainland can be collected on National Population and Health Science Data Sharing Platform. The data sets generated during and/or analyzed in this study are available from the corresponding authors on reasonable request.
