# Supplementary material for: The Effectiveness of National Expanded Program on Immunization With Hepatitis A Vaccines in the Chinese Mainland: Interrupted Time-Series Analysis
Source: JMIR Public Health Surveill. 2024 Feb 28;10:e53982. doi: 10.2196/53982 (PMC10938223; doi:10.2196/53982)
Supplement: Multimedia Appendix 1 [file publichealth_v10i1e53982_app1.pdf]

Supplemental Content

Supplemental methods **3**

Provincial-level-specific model 3

Effect estimators 3

Sensitivity analyses 4

Supplemental tables and figures **5**

Table S1. Details on the Expanded Program on Immunization information in the Chinese mainland. 5

Table S2. Hepatitis A cases before and after the intervention of the Expanded Program on Immunization. 7

Table S3. Values of quasi-Akaike information criterion in different modeling strategy. 8

Table S4. Description on hepatitis A public health emergencies in Chinese mainland. 9

Table S5. Autoregressive term of model residuals in the main analysis. 11

Table S6. Yearly hepatitis A cases and incidence among children aged 2-9 years in 7 regions of the Chinese mainland from 2005 to 2019. 12

Table S7. Excess risks of hepatitis A incidence associated with the Expanded Program on Immunization in the Chinese mainland. 13

Table S8. Average annual excess cases of hepatitis A associated with the Expanded Program on Immunization among the target population. 14

Table S9. Average annual excess incidence of hepatitis A associated with the Expanded Program on Immunization among the target population. 16

Table S10. Excess risks of hepatitis A incidence among children aged 2-9 years associated with the Expanded Program on Immunization in 7 regions of the Chinese mainland. 18

Table S11. Average annual excess incidence of hepatitis A associated with the Expanded Program on Immunization among the nontarget population. 20

Table S12. Average annual excess incidence of hepatitis A associated with the Expanded Program on Immunization among the whole population. 22

Figure S1. The partial auto-correlation functions of residuals from preliminary analyses for 30 provincial-level administrative divisions without an autoregressive term of residuals. 24

Figure S2. The partial auto-correlation functions of residuals from preliminary analyses for 30 provincial-level administrative divisions with an autoregressive term of residuals. 25

Figure S3. Monthly standardized hepatitis A incidence in the Chinese mainland. 26

Figure S4. Monthly incidence of hepatitis A among children aged 2-9 years in seven regions of Chinese mainland from 2005 to 2019. 27

Figure S5. Excess risks of hepatitis A among children aged 2-9 years associated with the Expanded Program on Immunization in 30 provincial-level administrative divisions. 28

Figure S6. Excess risks and excess incidence rates of hepatitis A among children aged 2-9 years in different subgroups. 30

Figure S7. Excess risks of hepatitis A incidence associated with the Expanded Program on Immunization in sensitivity analysis with the replacement of seasonality control. 31

Figure S8. Excess risks of hepatitis A incidence associated with the Expanded Program on Immunization in sensitivity analysis with the non-linear trend. 32

Figure S9. Excess risks of hepatitis A incidence associated with the Expanded Program on Immunization in sensitivity analysis with the transition period. 33

Supplemental methods

Provincial-level-specific model

We used a time-series quasi-Poisson regression to examine the province-specific effectiveness of the Expanded Program on Immunization (EPI). The formula of the model is as shown:

$$log[E(Y_{it})] = \alpha_i + offset(log(Population_{it})) + \beta_{i1}t + \beta_{i2}EPI_{it} + \beta_{i3}(t - Int_i) \times EPI_{it} + ns(Temperature_i, df = 3) + \gamma_i Month_{it} + \delta_i Indicator_{it} + \epsilon_\iota \varepsilon_{\iota,t-1}$$

where Y_{it} denotes the reported monthly number of hepatitis A cases in the PLADs i and the tth month under study (i = 1, 3,..., 31; t = 0, 1, 2, 3,..., 179); the logarithm of population was used as an offset; EPI_{it} is a dummy variable with 0 and 1 indicating the pre-intervention and post-intervention period, respectively; $t - Int_i$ is the time point when the EPI initiated in the PLADs i; $(t - Int_i) \times EPI_{it}$ indicates the time-varying change in the effectiveness of the EPI [1]; $ns(Temperature_i, df = 3)$ denotes natural cubic spline of monthly average temperature with 3 *dfs*; $Month_{it}$ is a categorical variable of calendar months; $Indicator_{it}$ denotes reported public health emergencies of hepatitis A (ie, five or more hepatitis A cases occurring within one week in the same collective unit according to historical reports or ≥20 cases occurring within one month) [2]; $\varepsilon_{\iota,t-1}$ is an autoregressive term of residuals at lag 1 to adjust for auto-correlation if necessary.

The models of the nontarget population and the whole population have the same structure as the target children model, except that interaction items are not considered.

Effect estimators

In the target population analysis, the provincial-specific excess risk (ER) of the hepatitis A incidence in the PLAD i and the time point t was expressed as: $\widehat{ER}_{it} = [exp(\widehat{\beta}_{i2} + \widehat{\beta}_{i3} \times t) - 1] \times 100\%$, and the corresponding 95% confidence intervals (CI) were estimated. By assuming the regression coefficients followed a multivariate normal distribution, the time-varying term also follows a multivariate normal distribution, which is as follows: $\widehat{\beta}_{i2} + \widehat{\beta}_{i3} \times t = TB \sim N_2(T\mu, T\Sigma T^T)$, where B is a joint normal distribution of $\widehat{\beta}_{i2}$ and $\widehat{\beta}_{i3}$, with a mean vector of μ and a covariance matrix of Σ; $T = [\mathbf{1}, t]$ is a constant matrix of t×2 dimensions. In non-target population analyses and whole population analyses, the ERs of the EPI was performed using the following formula: $\widehat{ER}_{it} = [exp(\widehat{\beta}_{i2}) - 1] \times 100\%$.

In the target population analysis, the excess incidence rate (EIR) in the province i was estimated as: $\widehat{EIR}_i = \sum_{t=t_0}^{t=t_1} [\widehat{Y}_{it}|(EPI_{it} = 1) - \widehat{Y}_{it}|(EPI_{it} = 0)]/Pop_i \times 100{,}000/Year_i$, where $\widehat{Y}_{it}|(EPI_{it} = 1)$ is the predicted number of cases in the province i and the post-intervention month t under the factual scenario that the EPI was implemented; $\widehat{Y}_{it}|(EPI_{it} = 0)$ is the predicted number of cases in province i under the counterfactual scenario that the EPI was not performed; Pop_i indicates the average target population size of province i in the post-intervention stage; $Year_i$ represents the number of post-intervention years under study for province i; t_0 and t_1 are the initial point of the EPI and the terminal point of the study period, respectively. Then, we use Monte Carlo simulation to estimate the 95% empirical CIs (eCIs) of EIR, because it is difficult to deduce the analytical formula for confidence intervals of EIRs [3]. Firstly, we took random samples $B^{(j)}$, with a mean of μ and a covariance of Σ, of the original parameters $\widehat{\beta}_{i2}$ and $\widehat{\beta}_{i3}$ derived from the regression model. Consequently, the distribution of EIR can be recalculated empirically. An 95% eCI could be defined with the 2.5th and 97.5th percentiles of the sampling distribution. For instance, 10,000 sets of $B^{(j)}$ (j = 1, 2,..., 10,000) were sampled from the multivariate normal distribution, calculating 10,000 sets of EIRs. The 2.5th and

97.5th percentiles (i.e. the 250th and 9,750th EIRs from the smallest to the largest) of these 10,000 EIRs were regarded as upper and lower bound of 95% eCI.

The calculation of EIR for the nontarget population model and the whole population model are identical to that for the target population model. The average population in the formula of EIR is based on the corresponding population group included in the model.

Sensitivity analyses

We undertook sensitivity analyses to examine the robustness of our results. First, we applied a natural cubic spline function with 3 *dfs* instead of calendar months to capture the seasonality of hepatitis A incidence. Second, we investigated the potentially nonlinear intervention effect over time by replacing $(t - Int_i) \times EPI_{it}$ with $ns(t - Int_i, df = 3) \times EPI_{it}$. Finally, we consider a transition period from the implementation of the EPI to 2010 and excluded this period from the analyses [4, 5], because the EPI was initially limited to several cities with the heavy burden of hepatitis A in several PLADs and did not cover the entire PLAD until 2010 [6].

Supplemental tables and figures

Table S1. Details on the Expanded Program on Immunization information in the Chinese mainland.

PLAD[a]	Pre-intervention period	Transition period[a]	Post-intervention period	Type of hepatitis A vaccine
Northern				
Beijing	2005.01-2008.12	/	2009.01-2018.12	Inactivated
Tianjin	2005.01-2008.08	2008.09-2008.12	2009.01-2018.12	Inactivated
Hebei	2005.01-2008.06	2008.07-2009.12	2009.01-2018.12	Live attenuated
Shanxi	2005.01-2008.07	/	2008.08-2018.12	Live attenuated
Inner Mongolia	2005.01-2008.07	2008.08-2009.12	2010.01-2018.12	Live attenuated
Northeast				
Liaoning	2005.01-2008.05	2008.06-2009.12	2010.01-2018.12	Live attenuated
Jilin	2005.01-2008.11	/	2008.12-2018.12	Live attenuated
Heilongjiang	2005.01-2008.06	/	2008.07-2018.12	Live attenuated
Eastern				
Shanghai	2005.01-2008.08	2008.09-2008.12	2009.01-2018.12	Inactivated
Jiangsu[b]	2005.01-2008.04	/	2008.05-2018.12	Inactivated, live attenuated
Zhejiang	2005.01-2008.04	2008.05-2009.12	2010.01-2018.12	Live attenuated
Anhui	2005.01-2008.03	2008.04-2009.12	2010.01-2018.12	Live attenuated
Fujian	2005.01-2008.12	2009.01-2009.12	2010.01-2018.12	Live attenuated
Jiangxi	2005.01-2008.06	2008.07-2008.12	2009.01-2018.12	Live attenuated
Shandong	2005.01-2008.04	2008.05-2010.04	2010.05-2018.12	Live attenuated
Central				
Henan	2005.01-2008.06	2008.07-2009.12	2010.01-2018.12	Live attenuated
Hubei	2005.01-2008.08	2008.09-2009.12	2010.01-2018.12	Live attenuated
Hunan	2005.01-2008.09	2008.10-2009.12	2010.01-2018.12	Live attenuated
Southern				
Guangdong	2005.01-2008.08	2008.09-2009.12	2010.01-2018.12	Live attenuated
Guangxi	2005.01-2008.03	2008.04-2009.12	2010.01-2018.12	Live attenuated
Hainan	2005.01-2008.06	2008.07-2009.12	2010.01-2018.12	Live attenuated
Southwest				
Chongqing	2005.01-2008.03	2008.04-2009.12	2010.01-2018.12	Live attenuated
Sichuan	2005.01-2008.06	2008.07-2009.12	2010.01-2018.12	Live attenuated
Guizhou	2005.01-2008.03	2008.04-2009.12	2010.01-2018.12	Live attenuated
Yunnan	2005.01-2008.06	2008.07-2009.12	2010.01-2018.12	Live attenuated
Tibet	2005.01-2008.06	2008.07-2009.12	2010.01-2018.12	Live attenuated
Northwest				
Shaanxi	2005.01-2008.06	2008.07-2010.06	2010.07-2018.12	Live attenuated
Gansu	2005.01-2008.09	2008.10-2009.12	2010.01-2018.12	Live attenuated
Qinghai	2005.01-2008.06	2008.07-2009.12	2010.01-2018.12	Live attenuated
Ningxia	2005.01-2008.06	2008.07-2009.12	2010.01-2018.12	Live attenuated
Xinjiang	2005.01-2008.06	2008.07-2009.12	2010.01-2018.12	Live attenuated

[a]The national document called for achieving and sustaining universal coverage of hepatitis A vaccines

for children by 2010. PLAD: provincial-level administrative division.

[b]Jiangsu integrated inactivated hepatitis A vaccines in 2008, but the type of vaccines changed to live attenuated vaccines at the end of 2011. Hence, when conducting subgroup analyses by types of hepatitis A, we considered three vaccine use scenarios (ie, inactivated vaccine, live attenuated vaccine, and both).

Table S2. Hepatitis A cases before and after the intervention of the Expanded Program on Immunization.

PLAD[a]	Pre-intervention cases	Post-intervention cases	Average annual cases before intervention	Average annual cases after intervention
Beijing	62	11	15.50	1.00
Tianjin	13	9	3.55	0.79
Hebei	1,120	384	320.00	33.39
Shanxi	269	151	75.07	13.23
Inner Mongolia	87	51	24.28	4.47
Liaoning	105	96	30.73	8.29
Jilin	44	34	11.23	3.07
Heilongjiang	47	34	13.43	2.96
Shanghai	60	22	16.36	1.94
Jiangsu	173	186	51.90	15.94
Zhejiang	509	334	152.70	28.63
Anhui	643	477	197.85	40.60
Fujian	126	85	31.50	7.73
Jiangxi	4,119	1,555	1,176.86	135.22
Shandong	244	194	73.20	16.63
Henan	7,256	5,567	2,176.80	477.17
Hubei	492	392	134.18	34.59
Hunan	959	169	255.73	15.02
Guangdong	331	462	90.27	40.76
Guangxi	478	421	147.08	35.83
Hainan	393	98	112.29	8.52
Chongqing	427	297	131.38	25.28
Sichuan	2,624	4,543	749.71	395.04
Guizhou	3,786	3,873	1,164.92	329.62
Yunnan	8,867	3,855	2,533.43	335.22
Tibet	583	770	145.75	70.00
Shaanxi	656	333	187.43	28.96
Gansu	4,720	2,981	1,258.67	264.98
Qinghai	890	1,311	242.73	115.68
Ningxia	1,879	826	512.45	72.88
Xinjiang	10,921	15,871	2,978.45	1,400.38

aPLAD: provincial-level administrative division.

Table S3. Values of quasi-Akaike information criterion in different modeling strategy.

PLAD[a]	*dfs*=3[b]	*dfs*=4[b]	*dfs*=5[b]	Without any covariate[c]
Beijing	222.10	222.17	225.07	194.53
Tianjin	/	/	/	/
Hebei	1,004.91	1,008.69	1,012.22	937.30
Shanxi	642.61	642.99	644.75	609.69
Inner Mongolia	405.23	407.88	403.33	370.37
Liaoning	484.57	487.69	489.39	451.18
Jilin	293.15	292.33	293.26	264.84
Heilongjiang	284.10	286.40	284.66	252.77
Shanghai	280.65	280.88	282.54	248.45
Jiangsu	645.38	651.42	641.23	604.51
Zhejiang	759.73	759.75	754.73	791.67
Anhui	902.88	907.10	893.29	931.82
Fujian	458.01	458.94	461.45	432.55
Jiangxi	1,230.93	1,235.36	1,249.46	1,414.78
Shandong	637.46	635.68	643.33	762.38
Henan	1,701.44	1,693.14	1,694.35	2,210.86
Hubei	993.49	1,001.45	1,006.02	1,074.63
Hunan	770.93	771.41	777.14	800.10
Guangdong	848.91	846.09	846.88	811.78
Guangxi	905.62	908.09	913.21	956.98
Hainan	486.35	486.09	487.88	451.36
Chongqing	867.23	871.10	876.32	809.00
Sichuan	1,539.76	1,522.55	1,533.24	1,478.94
Guizhou	1,651.70	1,703.76	1,762.51	2,315.55
Yunnan	1,576.97	1,575.21	1,572.27	2,623.16
Tibet	1,185.69	1,192.70	1,194.27	1,296.94
Shaanxi	847.26	851.14	856.22	791.28
Gansu	3,084.11	3,072.53	3,065.22	2,803.91
Qinghai	1,343.52	1,350.23	1,358.61	1,263.16
Ningxia	1,048.04	1,054.78	1,045.16	2,195.76
Xinjiang	4,105.68	4,109.52	4,456.02	9,823.81
Sum	31,208.41	31,287.09	31,724.04	39,974.03
Mean	1,040.28	1,042.90	1,057.47	1,332.47
SD[d]	821.65	821.70	868.65	1,760.01
Median	858.07	861.12	866.27	810.39

[a]PLAD: provincial-level administrative division.

[b]We replaced different *dfs* in the natural cubic spline of monthly average temperature.

[c]The model only contains 3 terms (ie, the time, the indicator variable of intervention, and the interaction term between the 2 variables mentioned above).

[d]SD: standard deviation.

Table S4. Description on hepatitis A public health emergencies in Chinese mainland.

PLAD[a]	Time	Spread of hepatitis A	Reference
Anhui	2011.04-2011.07	Elementary school	[7]
Zhejiang[b]	2007.10-2007.12	Elementary school and kindergarten	[8]
Jiangxi	2006.11-2006.12	University	[9]
	2007.01	Elementary school	[10]
Henan[c]	2006.10	Elementary school and middle school	[11]
	2007.01-2007.12	Prefecture	[12]
	2012.06-2012.11	Town	[13]
Hubei[d]	2005.03	Elementary school	[14]
Hunan	2006.03-2006.04	Elementary school	[15]
	2006.04-2006.07	Middle school	[16]
Hebei	2008.10-2008.12	Middle school	[17]
Guangxi	2006.11	Elementary school	[18]
	2007.05-2007.06	Town	[19]
	2010.03-2010.06	Elementary school	[20]
	2016.11-2016.12	Town	[21]
Shandong	2014.08	Elementary school and kindergarten	[22]
Sichuan	2007.03-2007.09	Prefecture	[23]
	2009.04-2009.05	Elementary school and middle school	[24]
Guizhou[e]	2007.05-2007.08	Elementary school and middle school	[25, 26]
	2008.03-2008.04	City	[27]
	2008.04-2008.05	Village	[28]
	2008.05-2008.06	Prefecture	[29]
	2010.03-2010.04	Prefecture	[30]
	2010.05-2010.06	Elementary school	[31]
	2010.08	Prefecture	[32]
Yunnan[f]	2008.04-2008.05	Middle school	[33]
Qinghai	2011.04	Elementary school	[34]
Ningxia[g]	2007.06-2007.12	Town	[35, 36]
	2008.11-2008.12	Kindergarten	[37]
Xinjiang	2005.11	Elementary school	[38]

[a]For public health emergencies without a clear date or a place of the occurrence, we consider that there was a hepatitis A public health emergency in that province in that year. PLAD: provincial-level administrative division.

[b]In Zhejiang, there were 1 public health emergency of hepatitis A in 2007 and one in 2009 [39].

[c] A total of 13 public health emergencies were reported from 2005 to 2020 in Henan [40, 41].

[d] A total of 6 public health emergencies were reported from 2005 to 2019 in Hubei, some of which were not included in the model for lack of the exact month or population [42].

[e] In 2009, there was a hepatitis A public health emergency in Zunyi City, Guizhou [43].
[f] A total of 26 public health emergencies were reported from 2004 to 2008 in Yunnan [44].
[g] In 2009, there was 1 hepatitis A public health emergency in Xiji County, Ningxia [36].

Table S5. Autoregressive term of model residuals in the main analysis.

Number (ι)	PLAD[a]	Lag[b]	Number (ι)	PLAD	Lag
1	Beijing	/	17	Hubei	1
2	Tianjin	/	18	Hunan	1
3	Hebei	1	19	Guangdong	1
4	Shanxi	/	20	Guangxi	1
5	Inner mongolia	/	21	Hainan	/
6	Liaoning	/	22	Chongqing	/
7	Jilin	/	23	Sichuan	1
8	Heilongjiang	/	24	Guizhou	/
9	Shanghai	/	25	Yunnan	1
10	Jiangsu	/	26	Tibet	1
11	Zhejiang	/	27	Shaanxi	/
12	Anhui	1	28	Gansu	1
13	Fujian	/	29	Qinghai	1
14	Jiangxi	/	30	Ningxia	/
15	Shandong	/	31	Xinjiang	1
16	Henan	1			

[a]PLAD: provincial-level administrative division.

[b]The lag equal to 1 represents that the model of the corresponding PLAD includes the residual regression term. / indicates the model without adding an autoregressive term of the residuals. The criterion for adding a residual lag term is whether the model residuals existed first-order partial autocorrelation in the main analysis.

Table S6. Yearly hepatitis A cases and incidence among children aged 2-9 years in seven regions of Chinese mainland from 2005 to 2019.

	Northeast		Northern		Northwest		Eastern		Central		Southern		Southwest	
Year	Cases	Incidence[a]	Cases	Incidence	Cases	Incidence	Cases	Incidence	Cases	Incidence	Cases	Incidence	Cases	Incidence
2005	80	0.96	2,134	14.33	4,948	42.99	1,700	5.42	2,153	11.18	366	2.09	3,594	15.03
2006	46	0.57	1,600	10.93	4,061	35.63	1,604	5.06	2,292	12.00	356	2.06	4,810	20.09
2007	36	0.47	1,167	8.15	7,896	72.14	1,986	6.38	3,570	18.75	360	2.11	6,036	26.48
2008	50	0.68	726	5.17	3,090	28.81	1,207	3.88	1,967	10.26	252	1.48	4,349	19.87
2009	27	0.38	455	3.28	1,641	15.41	763	2.45	1,881	9.95	225	1.32	3,310	15.64
2010	17	0.24	344	2.46	3,391	33.43	468	1.48	699	3.60	147	0.90	2,529	12.26
2011	20	0.29	278	1.95	2,633	27.29	314	0.97	864	4.30	115	0.73	972	4.87
2012	11	0.16	227	1.56	1,584	16.48	279	0.85	783	3.90	62	0.40	833	4.26
2013	5	0.07	181	1.24	1,542	16.02	107	0.32	307	1.49	41	0.27	814	4.16
2014	20	0.30	145	0.98	3,648	38.03	95	0.28	147	0.69	43	0.29	611	3.14
2015	13	0.20	105	0.71	2,081	21.28	44	0.13	55	0.25	26	0.16	507	2.62
2016	13	0.20	68	0.45	2,036	20.32	32	0.09	23	0.10	41	0.25	279	1.43
2017	8	0.12	43	0.28	1,065	10.46	34	0.10	28	0.12	45	0.27	354	1.80
2018	7	0.11	23	0.15	499	4.82	51	0.14	30	0.13	55	0.32	352	1.78
2019	7	0.11	23	0.15	273	2.68	43	0.12	36	0.16	49	0.29	275	1.40
Average	360	0.32	7,519	3.45	40,388	25.72	8,727	1.84	14,835	5.13	2,183	0.86	29,625	8.99

[a] The unit of the hepatitis A incidence is cases per 100,000 persons.

Table S7. Excess risks of hepatitis A incidence associated with the Expanded Program on Immunization in the Chinese mainland.

Intervention years after the EPI[a]	Main analysis		Sensitivity analysis – seasonality[b]		Sensitivity analysis – nonlinear trend[c]		Sensitivity analysis – transition period[d]	
	ER[e] (%)	95%CI	ER (%)	95%CI	ER (%)	95%CI	ER (%)	95%CI
1	–51.92	(–59.84 to –42.44)	–51.07	(–59.17 to –41.36)	–46.31	(–59.17 to –41.36)	–56.62	(–70.79 to –53.34)
2	–63.89	(–71.17 to –54.76)	–63.08	(–70.79 to –53.34)	–59.98	(–70.79 to –53.34)	–66.77	(–79.51 to –62.15)
3	–72.87	(–79.74 to –63.68)	–72.15	(–79.51 to –62.15)	–70.30	(–79.51 to –62.15)	–74.54	(–85.75 to –69.02)
4	–79.62	(–85.90 to –70.56)	–78.99	(–85.75 to –69.02)	–78.12	(–85.75 to –69.02)	–80.50	(–90.13 to –74.54)
5	–84.70	(–90.23 to –76.02)	–84.15	(–90.13 to –74.54)	–83.96	(–90.13 to –74.54)	–85.06	(–93.18 to –79.02)
6	–88.50	(–93.25 to –80.41)	–88.04	(–93.18 to –79.02)	–88.22	(–93.18 to –79.02)	–88.55	(–95.29 to –82.69)
7	–91.37	(–95.35 to –83.98)	–90.98	(–95.29 to –82.69)	–91.26	(–95.29 to –82.69)	–91.23	(–96.76 to –85.71)
8	–93.51	(–96.79 to –86.88)	–93.19	(–96.76 to –85.71)	–93.39	(–96.76 to –85.71)	–93.28	(–97.76 to –88.19)
9	–95.13	(–97.79 to –89.25)	–94.86	(–97.76 to –88.19)	–94.91	(–97.76 to –88.19)	–94.85	(–98.46 to –90.24)
10	–96.34	(–98.48 to –91.19)	–96.12	(–98.46 to –90.24)	–96.03	(–98.46 to –90.24)	–96.06	(–98.94 to –91.93)
11	–97.25	(–98.95 to –92.77)	–97.08	(–98.94 to –91.93)	–96.89	(–98.94 to –91.93)	/	/
Average	–80.77	(–85.86 to –72.92)	–80.25	(–85.66 to –71.68)	–79.05	(–84.68 to –70.41)	–80.45	(–86.48 to –71.19)
Heterogeneity of multivariate random–effects meta–analysis								
I^2 (%)	92.80		92.50		94.00		93.90	
P	<.001		<.001		<.001		<.001	

[a]EPI: Expanded Program on Immunization.

[b]A natural cubic spline function with 3 *dfs* to calendar months was applied to capture the seasonality of hepatitis A incidence.

[c]The potentially nonlinear intervention effect over time was investigated by applying a natural cubic spline function with 3 *dfs* to the time point when the EPI initiated in each PLADs.

[d]A transition period from the implementation of the EPI to 2010 was excluded this period from the analysis.

[e]ER: excess risk.

Table S8. Average annual excess cases of hepatitis A associated with the Expanded Program on Immunization among the target population.

Region	Excess cases (95% empirical CI)			
	1-5 years after the EPI[a]	6-11 years after the EPI	1-11 years after the EPI	From start of EPI to end of study period
Target population				
Chinese mainland	–16,991 (–17,545 to –16,031)	–49,307 (–50,026 to –47,379)	–34,618 (–35,197 to –33,215)	/
Northern	–198 (–220 to –158)	–225 (–238 to –150)	–213 (–229 to –158)	/
Beijing	–13 (–14 to –8)	–14 (–14 to –12)	–13 (–14 to –10)	–13 (–14 to –10)
Tianjin	/	/	/	/
Hebei	–159 (–179 to –127)	–165 (–172 to –145)	–162 (–175 to –138)	–163 (–176 to –139)
Shanxi	–0 (–9 to 18)	10 (0 to 64)	5 (–4 to 43)	5 (–4 to 44)
Inner Mongolia	–26 (–30 to –13)	–56 (–59 to –19)	–42 (–46 to –17)	–44 (–47 to –17)
Northeast	–5 (–14 to 25)	–5 (–12 to 99)	–5 (–13 to 65)	/
Liaoning	–0 (–7 to 19)	2 (–3 to 60)	1 (–5 to 41)	1 (–5 to 43)
Jilin	–6 (–10 to 7)	–9 (–11 to 15)	–8 (–10 to 11)	–8 (–10 to 11)
Heilongjiang	2 (–2 to 20)	2 (–0 to 67)	2 (–1 to 46)	2 (–1 to 50)
Eastern	–1,763 (–1,835 to –1,649)	–7,774 (–7,793 to –7,688)	–5,042 (–5,082 to –4,952)	/
Shanghai	–9 (–11 to –1)	–9 (–10 to 6)	–9 (–10 to 3)	–9 (–10 to 3)
Jiangsu	–271 (–286 to –235)	–2,637 (–2,642 to –2,601)	–1,562 (–1,571 to –1,526)	–2,002 (–2,011 to –1,966)
Zhejiang	–477 (–499 to –439)	–3,056 (–3,062 to –3,029)	–1,883 (–1,897 to –1,852)	–2,266 (–2,279 to –2,236)
Anhui	–458 (–486 to –409)	–1,422 (–1,433 to –1,384)	–984 (–1,002 to –942)	–1,160 (–1,178 to –1,118)
Fujian	–55 (–60 to –44)	–162 (–166 to –144)	–113 (–118 to –99)	–113 (–118 to –99)
Jiangxi	–495 (–557 to –414)	–489 (–495 to –476)	–277 (–309 to –232)	–486 (–516 to –445)
Shandong	1 (–13 to 33)	–1 (–9 to 41)	–0 (–10 to 37)	–0 (–10 to 36)

[a]EPI: Expanded Program on Immunization.

Table S8. Average annual excess cases of hepatitis A associated with the Expanded Program on Immunization among the target population. *continued*.

Region	Excess cases (95% empirical CI)			
	1-5 years after the EPI[a]	6-11 years after the EPI	1-11 years after the EPI	From start of EPI to end of study period
Target population				
Central	–2,353 (–2,624 to –1,962)	–2,954 (–2,987 to –2,876)	–2,680 (–2,820 to –2,463)	/
Henan	–2,255 (–2,527 to –1,868)	–2,829 (–2,861 to –2,765)	–2,568 (–2,709 to –2,360)	–2,627 (–2,760 to –2,431)
Hubei	–26 (–54 to 29)	–111 (–119 to –75)	–72 (–89 to –28)	–73 (–89 to –29)
Hunan	–72 (–79 to –61)	–13 (–20 to 4)	–40 (–47 to –26)	–39 (–46 to –25)
Southern	–198 (–237 to –126)	–574 (–599 to –475)	–403 (–434 to –318)	/
Guangdong	–47 (–71 to –2)	–123 (–142 to –51)	–88 (–110 to –29)	–91 (–112 to –31)
Guangxi	–148 (–176 to –92)	–451 (–464 to –388)	–313 (–333 to –255)	–345 (–364 to –286)
Hainan	–3 (–10 to 10)	–0 (–2 to 5)	–2 (–5 to 7)	–1 (–5 to 7)
Southwest	–6,136 (–6,464 to –5,545)	–19,265 (–19,412 to –18,785)	–13,297 (–13,501 to –12,820)	/
Chongqing	–131 (–152 to –88)	–268 (–275 to –236)	–206 (–219 to –170)	–219 (–232 to –184)
Sichuan	–415 (–533 to –251)	–1,173 (–1,306 to –919)	–828 (–953 to –617)	–868 (–992 to –652)
Guizhou	–289 (–566 to 234)	–267 (–287 to –101)	–277 (–413 to 45)	–275 (–403 to 33)
Yunnan	–5,204 (–5,332 to –5,048)	–17,314 (–17,335 to –17,281)	–11,809 (–11,878 to –11,722)	–18,516 (–18,582 to –18,430)
Tibet	–98 (–150 to 32)	–243 (–293 to 93)	–177 (–228 to 64)	–184 (–236 to 67)
Northwest	–6,338 (–6,720 to –5,767)	–18,510 (–19,312 to –16,786)	–12,977 (–13,573 to –11,791)	/
Shaanxi	–176 (–196 to –142)	–365 (–368 to –353)	–279 (–290 to –258)	–294 (–305 to –274)
Gansu	–1,189 (–1,307 to –1,030)	–1,899 (–1,928 to –1,844)	–1,576 (–1,645 to –1,475)	–1,584 (–1,651 to –1,485)
Qinghai	–939 (–1,007 to –825)	–3,656 (–3,688 to –3,569)	–2,421 (–2,469 to –2,323)	–2,617 (–2,664 to –2,520)
Ningxia	–143 (–196 to –52)	–47 (–52 to –8)	–91 (–117 to –29)	–90 (–115 to –29)
Xinjiang	–3,891 (–4,240 to –3,369)	–12,543 (–13,347 to –10,823)	–8,611 (–9,202 to –7,449)	–8,774 (–9,365 to –7,595)

[a]EPI: Expanded Program on Immunization..

Table S9. Average annual excess incidence of hepatitis A associated with the Expanded Program on Immunization among the target population.

Region	Excess incidence rate (95% empirical CI)			
	1-5 years after the EPI[a]	6-11 years after the EPI	1-11 years after the EPI	From start of EPI to end of study period
Target population				
Chinese mainland	–14.31 (–14.78 to –13.50)	–39.90 (–40.48 to –38.34)	–28.52 (–29.00 to –27.37)	/
Northern	–1.47 (–1.64 to –1.18)	–1.58 (–1.67 to –1.06)	–1.53 (–1.65 to –1.14)	/
Beijing	–1.27 (–1.41 to –0.84)	–1.08 (–1.09 to –0.92)	–1.16 (–1.21 to –0.90)	–1.16 (–1.21 to –0.90)
Tianjin	/	/	/	/
Hebei	–2.29 (–2.58 to –1.83)	–2.13 (–2.22 to –1.88)	–2.20 (–2.37 to –1.87)	–2.21 (–2.37 to –1.88)
Shanxi	–0.01 (–0.25 to 0.53)	0.29 (0.00 to 1.95)	0.15 (–0.12 to 1.27)	0.15 (–0.11 to 1.33)
Inner Mongolia	–1.26 (–1.45 to –0.66)	–2.87 (–3.03 to –0.97)	–2.12 (–2.29 to –0.83)	–2.20 (–2.37 to –0.85)
Northeast	–0.07 (–0.20 to 0.37)	–0.08 (–0.19 to 1.49)	–0.07 (–0.19 to 0.96)	/
Liaoning	–0.01 (–0.25 to 0.70)	0.09 (–0.13 to 2.33)	0.04 (–0.19 to 1.57)	0.05 (–0.18 to 1.64)
Jilin	–0.36 (–0.55 to 0.39)	–0.51 (–0.60 to 0.86)	–0.44 (–0.58 to 0.63)	–0.44 (–0.57 to 0.63)
Heilongjiang	0.07 (–0.06 to 0.80)	0.07 (–0.01 to 2.97)	0.07 (–0.04 to 1.94)	0.07 (–0.03 to 2.12)
Eastern	–5.50 (–5.72 to –5.14)	–22.37 (–22.42 to –22.12)	–15.04 (–15.15 to –14.77)	/
Shanghai	–0.80 (–1.00 to –0.05)	–0.70 (–0.77 to 0.51)	–0.74 (–0.87 to 0.23)	–0.73 (–0.85 to 0.24)
Jiangsu	–5.16 (–5.45 to –4.48)	–44.08 (–44.16 to –43.48)	–27.64 (–27.81 to –27.00)	–35.32 (–35.48 to –34.69)
Zhejiang	–13.09 (–13.69 to –12.04)	–83.95 (–84.14 to –83.21)	–51.73 (–52.10 to –50.86)	–61.92 (–62.27 to –61.08)
Anhui	–7.82 (–8.30 to –6.98)	–22.53 (–22.71 to –21.93)	–16.12 (–16.42 to –15.43)	–18.96 (–19.26 to –18.27)
Fujian	–1.73 (–1.90 to –1.37)	–4.61 (–4.72 to –4.12)	–3.37 (–3.51 to –2.95)	–3.37 (–3.51 to –2.95)
Jiangxi	–9.76 (–10.97 to –8.16)	–9.30 (–9.43 to –9.06)	–9.50 (–10.11 to –8.68)	–9.40 (–9.98 to –8.62)
Shandong	0.01 (–0.16 to 0.42)	–0.01 (–0.10 to 0.47)	–0.00 (–0.12 to 0.43)	–0.00 (–0.12 to 0.42)

[a]EPI: Expanded Program on Immunization.

Table S9. Average annual excess incidence of hepatitis A associated with the Expanded Program on Immunization among the target population. *continued.*

Region	Excess incidence rate (95% empirical CI)			
	1-5 years after the EPI[a]	6-11 years after the EPI	1-11 years after the EPI	From start of EPI to end of study period
Target population				
Central	–11.95 (–13.32 to –9.96)	–13.35 (–13.50 to –13.00)	–12.75 (–13.42 to –11.71)	/
Henan	–21.73 (–24.35 to –18.00)	–25.44 (–25.73 to –24.87)	–23.82 (–25.12 to –21.89)	–24.35 (–25.58 to –22.53)
Hubei	–0.83 (–1.75 to 0.94)	–2.54 (–2.71 to –1.71)	–1.91 (–2.35 to –0.73)	–1.91 (–2.34 to –0.76)
Hunan	–1.16 (–1.27 to –0.98)	–0.20 (–0.30 to 0.06)	–0.62 (–0.72 to –0.41)	–0.61 (–0.71 to –0.39)
Southern	–1.23 (–1.47 to –0.78)	–3.53 (–3.68 to –2.92)	–2.49 (–2.68 to –1.97)	/
Guangdong	–0.49 (–0.74 to –0.02)	–1.26 (–1.46 to –0.52)	–0.91 (–1.13 to –0.30)	–0.94 (–1.15 to –0.32)
Guangxi	–2.67 (–3.19 to –1.66)	–8.03 (–8.25 to –6.91)	–5.62 (–5.97 to –4.56)	–6.17 (–6.51 to –5.10)
Hainan	–0.32 (–1.10 to 1.09)	–0.04 (–0.17 to 0.49)	–0.17 (–0.58 to 0.72)	–0.16 (–0.56 to 0.70)
Southwest	–30.01 (–31.61 to –27.12)	–98.50 (–99.25 to –96.05)	–66.61 (–67.63 to –64.22)	/
Chongqing	–4.75 (–5.53 to –3.21)	–10.00 (–10.25 to –8.80)	–7.58 (–8.07 to –6.26)	–8.08 (–8.54 to –6.78)
Sichuan	–5.69 (–7.30 to –3.44)	–16.59 (–18.47 to –13.00)	–11.55 (–13.28 to –8.60)	–12.09 (–13.83 to –9.09)
Guizhou	–6.19 (–12.13 to 5.02)	–6.38 (–6.86 to –2.41)	–6.29 (–9.39 to 1.02)	–6.27 (–9.18 to 0.75)
Yunnan	–102.51 (–105.04 to –99.45)	–357.23 (–357.66 to –356.54)	–238.52 (–239.90 to –236.76)	–374.84 (–376.19 to –373.11)
Tibet	–14.69 (–22.46 to 4.80)	–31.39 (–37.93 to 12.01)	–24.39 (–31.43 to 8.78)	–25.63 (–32.84 to 9.36)
Northwest	–63.25 (–67.06 to –57.55)	–185.28 (–193.32 to –168.03)	–129.72 (–135.67 to –117.86)	/
Shaanxi	–5.63 (–6.29 to –4.56)	–12.60 (–12.73 to –12.20)	–9.30 (–9.68 to –8.61)	–9.83 (–10.19 to –9.16)
Gansu	–46.19 (–50.78 to –40.01)	–78.68 (–79.88 to –76.42)	–63.39 (–66.14 to –59.33)	–63.73 (–66.43 to –59.75)
Qinghai	–79.69 (–85.49 to –70.05)	–321.63 (–324.48 to –313.98)	–209.50 (–213.69 to –201.04)	–229.07 (–233.24 to –220.58)
Ningxia	–19.70 (–26.95 to –7.16)	–6.67 (–7.35 to –1.19)	–12.70 (–16.37 to –4.09)	–12.54 (–16.10 to –4.01)
Xinjiang	–160.76 (–175.17 to –139.19)	–440.96 (–469.23 to –380.48)	–324.70 (–347.01 to –280.89)	–328.79 (–350.94 to –284.63)

[a]EPI: Expanded Program on Immunization..

Table S10. Excess risks of hepatitis A incidence among children aged 2-9 years associated with the Expanded Program on Immunization in 7 regions of the Chinese mainland.

Intervention years	Northeast		Northern		Northwest		Eastern	
after the EPI[a]	ER[b] (%)	95% CI	ER (%)	95% CI	ER (%)	95% CI	ER (%)	95% CI
1	–17.26	(–56.04 to 55.73)	–60.88	(–75.49 to –37.54)	–56.44	(–64.73 to –46.20)	–60.12	(–73.13 to –40.83)
2	–13.95	(–62.27 to 96.23)	–66.27	(–84.59 to –26.16)	–70.18	(–74.90 to –64.56)	–73.36	(–84.13 to –55.26)
3	–10.51	(–68.16 to 151.5)	–70.92	(–90.48 to –11.22)	–79.58	(–83.56 to –74.64)	–82.20	(–90.80 to –65.55)
4	–6.93	(–73.36 to 225.09)	–74.93	(–94.14 to 7.29)	–86.02	(–89.75 to –80.94)	–88.11	(–94.71 to –73.24)
5	–3.21	(–77.81 to 322.21)	–78.39	(–96.41 to 29.98)	–90.43	(–93.72 to –85.42)	–92.05	(–96.98 to –79.12)
6	0.66	(–81.58 to 449.93)	–81.37	(–97.80 to 57.64)	–93.45	(–96.18 to –88.77)	–94.69	(–98.28 to –83.66)
7	4.68	(–84.73 to 617.63)	–83.94	(–98.65 to 91.33)	–95.52	(–97.68 to –91.32)	–96.45	(–99.02 to –87.19)
8	8.87	(–87.36 to 837.66)	–86.15	(–99.17 to 132.33)	–96.93	(–98.60 to –93.28)	–97.63	(–99.44 to –89.94)
9	13.22	(–89.55 to 1126.27)	–88.06	(–99.49 to 182.2)	–97.90	(–99.15 to –94.80)	–98.42	(–99.68 to –92.10)
10	17.75	(–91.36 to 1504.79)	–89.71	(–99.69 to 242.84)	–98.56	(–99.49 to –95.97)	–98.94	(–99.82 to –93.79)
11	22.46	(–92.86 to 2001.23)	–91.13	(–99.81 to 316.58)	–99.02	(–99.69 to –96.87)	–99.29	(–99.90 to –95.12)
Average	–0.37	(–76.90 to 585.36)	–77.77	(–92.75 to 74.00)	–85.21	(–88.83 to –79.83)	–86.93	(–92.50 to –74.81)
Heterogeneity of multivariate random–effects meta–analysis								
I^2 (%)	1.00		84.90		93.60		81.50	
P	.40		<.001		<.001		<.001	

[a]EPI: Expanded Program on Immunization.

[b]ER: excess risk.

Table S10. Excess risks of hepatitis A incidence among children aged 2-9 years associated with the Expanded Program on Immunization in 7 regions of the Chinese mainland. *continued.*

Intervention years after the EPI[a]	Central		Southern		Southwest	
	ER[b] (%)	95% CI	ER (%)	95% CI	ER (%)	95% CI
1	–51.39	(–81.24 to 25.96)	–31.33	(–53.65 to 1.73)	–44.75	(–66.75 to –8.20)
2	–63.17	(–79.74 to –33.06)	–43.49	(–67.89 to –0.58)	–60.57	(–80.11 to –21.84)
3	–72.10	(–81.20 to –58.60)	–53.50	(–77.91 to –2.12)	–71.87	(–88.16 to –33.13)
4	–78.86	(–87.80 to –63.37)	–61.74	(–84.86 to –3.34)	–79.92	(–92.97 to –42.66)
5	–83.99	(–93.44 to –60.90)	–68.52	(–89.63 to –4.39)	–85.67	(–95.83 to –50.77)
6	–87.87	(–96.63 to –56.35)	–74.09	(–92.91 to –5.35)	–89.78	(–97.53 to –57.71)
7	–90.81	(–98.29 to –50.58)	–78.68	(–95.15 to –6.24)	–92.70	(–98.54 to –63.64)
8	–93.04	(–99.14 to –43.71)	–82.46	(–96.69 to –7.09)	–94.79	(–99.13 to –68.73)
9	–94.72	(–99.57 to –35.68)	–85.57	(–97.74 to –7.91)	–96.28	(–99.49 to –73.11)
10	–96.00	(–99.78 to –26.38)	–88.12	(–98.45 to –8.70)	–97.35	(–99.70 to –76.86)
11	–96.97	(–99.89 to –15.64)	–90.23	(–98.94 to –9.48)	–98.11	(–99.82 to –80.09)
Average	–80.21	(–91.72 to –32.63)	–65.92	(–84.19 to –4.24)	–79.94	(–90.47 to –48.84)
Heterogeneity of multivariate random–effects meta–analysis						
I^2 (%)	98.20		49.80		94.50	
P	<.001		.09		<.001	

[a]EPI: Expanded Program on Immunization.

[b]ER: excess risk.

Table S11. Average annual excess incidence of hepatitis A associated with the Expanded Program on Immunization among the nontarget population.

Region	Excess incidence rate (95% empirical CI)			
	1-5 years after the EPI[a]	6-11 years after the EPI	1-11 years after the EPI	From start of EPI to end of study period
Non–target population				
Chinese mainland	–0.84 (–0.89 to –0.79)	–0.73 (–0.77 to –0.68)	–0.78 (–0.82 to –0.73)	/
Beijing	–0.96 (–1.10 to –0.79)	–0.93 (–1.06 to –0.76)	–0.94 (–1.08 to –0.77)	–0.94 (–1.08 to –0.77)
Tianjin	–0.45 (–0.54 to –0.33)	–0.65 (–0.77 to –0.47)	–0.57 (–0.67 to –0.41)	–0.57 (–0.68 to –0.41)
Hebei	–0.49 (–0.58 to –0.38)	–0.34 (–0.40 to –0.27)	–0.41 (–0.48 to –0.32)	–0.40 (–0.48 to –0.32)
Shanxi	–1.78 (–2.03 to –1.50)	–2.48 (–2.84 to –2.09)	–2.17 (–2.48 to –1.83)	–2.20 (–2.52 to –1.86)
Inner Mongolia	–1.21 (–1.42 to –0.97)	–0.77 (–0.90 to –0.62)	–0.97 (–1.13 to –0.77)	–0.96 (–1.12 to –0.77)
Liaoning	–3.11 (–3.67 to –2.39)	–4.76 (–5.61 to –3.65)	–4.01 (–4.73 to –3.08)	–4.05 (–4.77 to –3.10)
Jilin	–0.70 (–0.87 to –0.51)	–0.46 (–0.57 to –0.33)	–0.57 (–0.71 to –0.41)	–0.57 (–0.71 to –0.41)
Heilongjiang	–0.80 (–0.90 to –0.67)	–0.58 (–0.65 to –0.48)	–0.68 (–0.77 to –0.57)	–0.67 (–0.76 to –0.57)
Shanghai	–1.46 (–1.62 to –1.27)	–1.79 (–1.99 to –1.56)	–1.65 (–1.82 to –1.43)	–1.65 (–1.83 to –1.44)
Jiangsu	–0.69 (–0.80 to –0.56)	–0.38 (–0.44 to –0.31)	–0.52 (–0.61 to –0.42)	–0.51 (–0.60 to –0.41)
Zhejiang	–1.41 (–1.56 to –1.25)	–0.84 (–0.93 to –0.74)	–1.09 (–1.21 to –0.97)	–1.07 (–1.18 to –0.95)
Anhui	–0.44 (–0.58 to –0.29)	–0.26 (–0.34 to –0.17)	–0.34 (–0.45 to –0.22)	–0.33 (–0.44 to –0.22)
Fujian	–0.41 (–0.61 to –0.20)	–0.25 (–0.37 to –0.12)	–0.32 (–0.48 to –0.15)	–0.32 (–0.48 to –0.15)
Jiangxi	–1.18 (–1.35 to –0.99)	–0.58 (–0.66 to –0.49)	–0.85 (–0.97 to –0.71)	–0.83 (–0.95 to –0.70)
Shandong	–0.51 (–0.56 to –0.44)	–0.52 (–0.58 to –0.46)	–0.52 (–0.57 to –0.45)	–0.52 (–0.57 to –0.45)

[a]EPI: Expanded Program on Immunization.

Table S11. Average annual excess incidence of hepatitis A associated with the Expanded Program on Immunization among the nontarget population. *continued*.

Region	Excess incidence rate (95% CI)			
	1-5 years after the EPI[a]	6-11 years after the EPI	1-11 years after the EPI	From start of EPI to end of study period
Non–target population				
Henan	0.68 (0.50 to 0.88)	0.15 (0.11 to 0.19)	0.39 (0.29 to 0.51)	0.37 (0.28 to 0.48)
Hubei	–0.85 (–1.02 to –0.68)	–0.58 (–0.70 to –0.46)	–0.71 (–0.85 to –0.56)	–0.70 (–0.84 to –0.55)
Hunan	–0.61 (–0.76 to –0.44)	–0.38 (–0.47 to –0.27)	–0.48 (–0.60 to –0.35)	–0.48 (–0.60 to –0.34)
Guangdong	–0.46 (–0.57 to –0.34)	–0.47 (–0.58 to –0.35)	–0.47 (–0.58 to –0.35)	–0.47 (–0.58 to –0.35)
Guangxi	–0.24 (–0.51 to 0.07)	–0.14 (–0.30 to 0.04)	–0.19 (–0.39 to 0.05)	–0.18 (–0.39 to 0.05)
Hainan	–1.22 (–1.58 to –0.80)	–0.49 (–0.63 to –0.32)	–0.81 (–1.05 to –0.53)	–0.79 (–1.02 to –0.52)
Chongqing	–2.36 (–2.69 to –2.01)	–1.50 (–1.72 to –1.28)	–1.88 (–2.15 to –1.60)	–1.84 (–2.10 to –1.56)
Sichuan	–1.08 (–1.36 to –0.78)	–0.60 (–0.75 to –0.44)	–0.81 (–1.02 to –0.59)	–0.80 (–1.01 to –0.58)
Guizhou	–0.84 (–1.54 to –0.08)	–0.14 (–0.26 to –0.01)	–0.45 (–0.83 to –0.04)	–0.43 (–0.79 to –0.04)
Yunnan	–1.41 (–1.98 to –0.74)	–0.75 (–1.06 to –0.39)	–1.04 (–1.46 to –0.55)	–1.02 (–1.43 to –0.54)
Tibet	–1.68 (–2.81 to –0.25)	–1.08 (–1.81 to –0.16)	–1.35 (–2.25 to –0.20)	–1.37 (–2.29 to –0.21)
Shaanxi	–0.20 (–0.37 to –0.01)	–0.09 (–0.18 to –0.00)	–0.14 (–0.26 to –0.01)	–0.14 (–0.26 to –0.01)
Gansu	0.46 (–0.26 to 1.27)	0.15 (–0.08 to 0.41)	0.29 (–0.16 to 0.79)	0.29 (–0.16 to 0.78)
Qinghai	–1.11 (–2.26 to 0.18)	–0.75 (–1.53 to 0.12)	–0.91 (–1.85 to 0.15)	–0.90 (–1.84 to 0.15)
Ningxia	–2.60 (–3.39 to –1.69)	–1.06 (–1.39 to –0.69)	–1.74 (–2.26 to –1.13)	–1.71 (–2.23 to –1.11)
Xinjiang	–5.18 (–5.88 to –4.39)	–4.58 (–5.21 to –3.88)	–4.84 (–5.50 to –4.10)	–4.77 (–5.43 to –4.04)

[a]EPI: Expanded Program on Immunization.

Table S12. Average annual excess incidence of hepatitis A associated with the Expanded Program on Immunization among the whole population.

Region	Excess incidence rate (95% empirical CI)			
	1-5 years after the EPI[a]	6-11 years after the EPI	1-11 years after the EPI	From start of EPI to end of study period
Whole population				
Chinese mainland	–0.87 (–0.93 to –0.81)	–0.80 (–0.84 to –0.75)	–0.83 (–0.88 to –0.78)	/
Beijing	–0.96 (–1.09 to –0.80)	–0.91 (–1.03 to –0.75)	–0.93 (–1.05 to –0.77)	–0.93 (–1.05 to –0.77)
Tianjin	–0.44 (–0.52 to –0.33)	–0.64 (–0.75 to –0.47)	–0.56 (–0.66 to –0.41)	–0.56 (–0.66 to –0.42)
Hebei	–0.66 (–0.75 to –0.56)	–0.43 (–0.49 to –0.37)	–0.53 (–0.61 to –0.45)	–0.52 (–0.60 to –0.44)
Shanxi	–1.72 (–1.97 to –1.46)	–2.40 (–2.74 to –2.03)	–2.10 (–2.39 to –1.78)	–2.13 (–2.43 to –1.81)
Inner Mongolia	–1.12 (–1.32 to –0.89)	–0.72 (–0.84 to –0.57)	–0.90 (–1.06 to –0.72)	–0.89 (–1.05 to –0.71)
Liaoning	–2.89 (–3.41 to –2.21)	–4.42 (–5.22 to –3.38)	–3.72 (–4.40 to –2.85)	–3.76 (–4.43 to –2.87)
Jilin	–0.66 (–0.82 to –0.47)	–0.43 (–0.53 to –0.31)	–0.53 (–0.66 to –0.38)	–0.53 (–0.66 to –0.38)
Heilongjiang	–0.75 (–0.85 to –0.63)	–0.54 (–0.61 to –0.45)	–0.63 (–0.72 to –0.53)	–0.63 (–0.71 to –0.53)
Shanghai	–1.42 (–1.57 to –1.24)	–1.72 (–1.90 to –1.50)	–1.59 (–1.76 to –1.39)	–1.60 (–1.76 to –1.40)
Jiangsu	–0.64 (–0.75 to –0.51)	–0.34 (–0.40 to –0.27)	–0.47 (–0.56 to –0.38)	–0.47 (–0.55 to –0.38)
Zhejiang	–1.36 (–1.50 to –1.20)	–0.77 (–0.85 to –0.68)	–1.03 (–1.14 to –0.91)	–1.01 (–1.12 to –0.89)
Anhui	–0.48 (–0.62 to –0.34)	–0.27 (–0.34 to –0.19)	–0.36 (–0.47 to –0.25)	–0.36 (–0.45 to –0.25)
Fujian	–0.40 (–0.58 to –0.20)	–0.24 (–0.36 to –0.12)	–0.31 (–0.46 to –0.16)	–0.31 (–0.46 to –0.16)
Jiangxi	–1.06 (–1.26 to –0.85)	–0.36 (–0.43 to –0.29)	–0.68 (–0.80 to –0.54)	–0.66 (–0.78 to –0.53)
Shandong	–0.48 (–0.53 to –0.42)	–0.48 (–0.53 to –0.42)	–0.48 (–0.53 to –0.42)	–0.47 (–0.53 to –0.42)

[a]EPI: Expanded Program on Immunization.

Table S12. Average annual excess incidence of hepatitis A associated with the Expanded Program on Immunization among the whole population. *continued.*

Region	Excess incidence rate (95% empirical CI)			
	1-5 years after the EPI[a]	6-11 years after the EPI	1-11 years after the EPI	From start of EPI to end of study period
Whole population				
Henan	1.27 (0.98 to 1.59)	0.21 (0.16 to 0.26)	0.69 (0.53 to 0.86)	0.65 (0.50 to 0.82)
Hubei	–0.77 (–0.95 to –0.57)	–0.49 (–0.61 to –0.37)	–0.62 (–0.76 to –0.46)	–0.61 (–0.75 to –0.45)
Hunan	–0.71 (–0.86 to –0.55)	–0.44 (–0.53 to –0.34)	–0.56 (–0.68 to –0.43)	–0.56 (–0.67 to –0.43)
Guangdong	–0.40 (–0.50 to –0.29)	–0.40 (–0.51 to –0.29)	–0.40 (–0.50 to –0.29)	–0.40 (–0.50 to –0.29)
Guangxi	–0.22 (–0.48 to 0.07)	–0.13 (–0.28 to 0.04)	–0.17 (–0.37 to 0.06)	–0.17 (–0.36 to 0.06)
Hainan	–1.26 (–1.59 to –0.89)	–0.47 (–0.59 to –0.33)	–0.82 (–1.03 to –0.57)	–0.79 (–1.00 to –0.56)
Chongqing	–2.18 (–2.49 to –1.85)	–1.37 (–1.56 to –1.16)	–1.73 (–1.97 to –1.46)	–1.69 (–1.93 to –1.43)
Sichuan	–0.97 (–1.25 to –0.69)	–0.55 (–0.71 to –0.39)	–0.74 (–0.95 to –0.52)	–0.73 (–0.93 to –0.51)
Guizhou	–0.24 (–1.03 to 0.67)	–0.03 (–0.13 to 0.09)	–0.13 (–0.54 to 0.35)	–0.12 (–0.51 to 0.33)
Yunnan	–1.62 (–2.29 to –0.88)	–0.68 (–0.96 to –0.37)	–1.10 (–1.55 to –0.60)	–1.07 (–1.52 to –0.58)
Tibet	–0.27 (–1.91 to 1.74)	–0.16 (–1.15 to 1.04)	–0.21 (–1.48 to 1.34)	–0.21 (–1.51 to 1.37)
Shaanxi	–0.23 (–0.40 to –0.04)	–0.10 (–0.18 to –0.02)	–0.16 (–0.28 to –0.03)	–0.16 (–0.27 to –0.03)
Gansu	0.34 (–0.56 to 1.31)	0.10 (–0.16 to 0.37)	0.20 (–0.34 to 0.79)	0.20 (–0.33 to 0.78)
Qinghai	0.17 (–1.12 to 1.59)	0.09 (–0.63 to 0.89)	0.13 (–0.85 to 1.20)	0.13 (–0.84 to 1.20)
Ningxia	–3.66 (–4.66 to –2.48)	–1.03 (–1.31 to –0.70)	–2.19 (–2.78 to –1.48)	–2.15 (–2.73 to –1.45)
Xinjiang	–11.85 (–13.28 to –10.26)	–11.93 (–13.36 to –10.32)	–11.90 (–13.32 to –10.29)	–11.73 (–13.14 to –10.15)

[a]EPI: Expanded Program on Immunization.

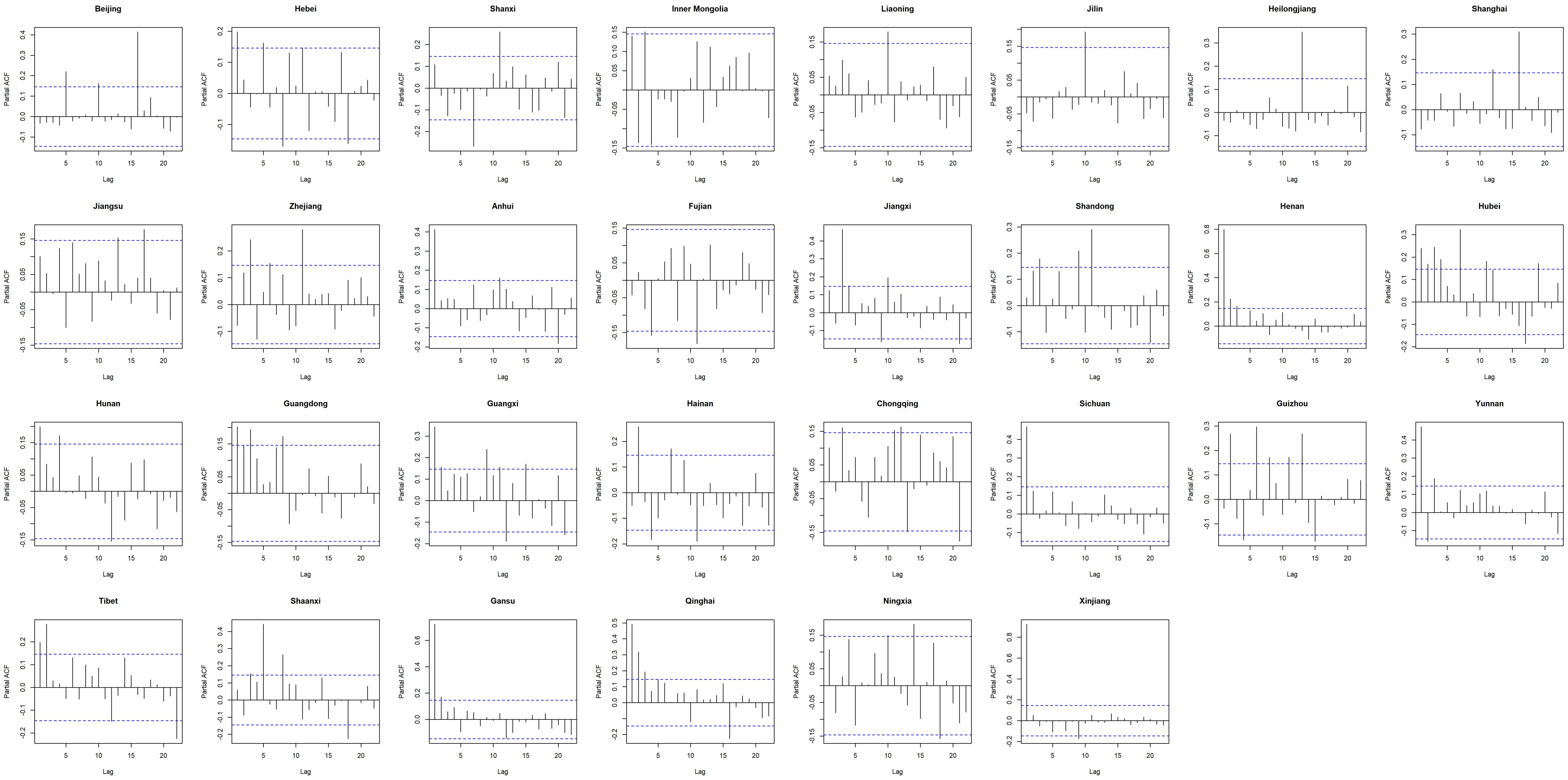

Figure S1. The partial auto-correlation functions of residuals from preliminary analyses for 30 provincial-level administrative divisions without an autoregressive term of residuals.

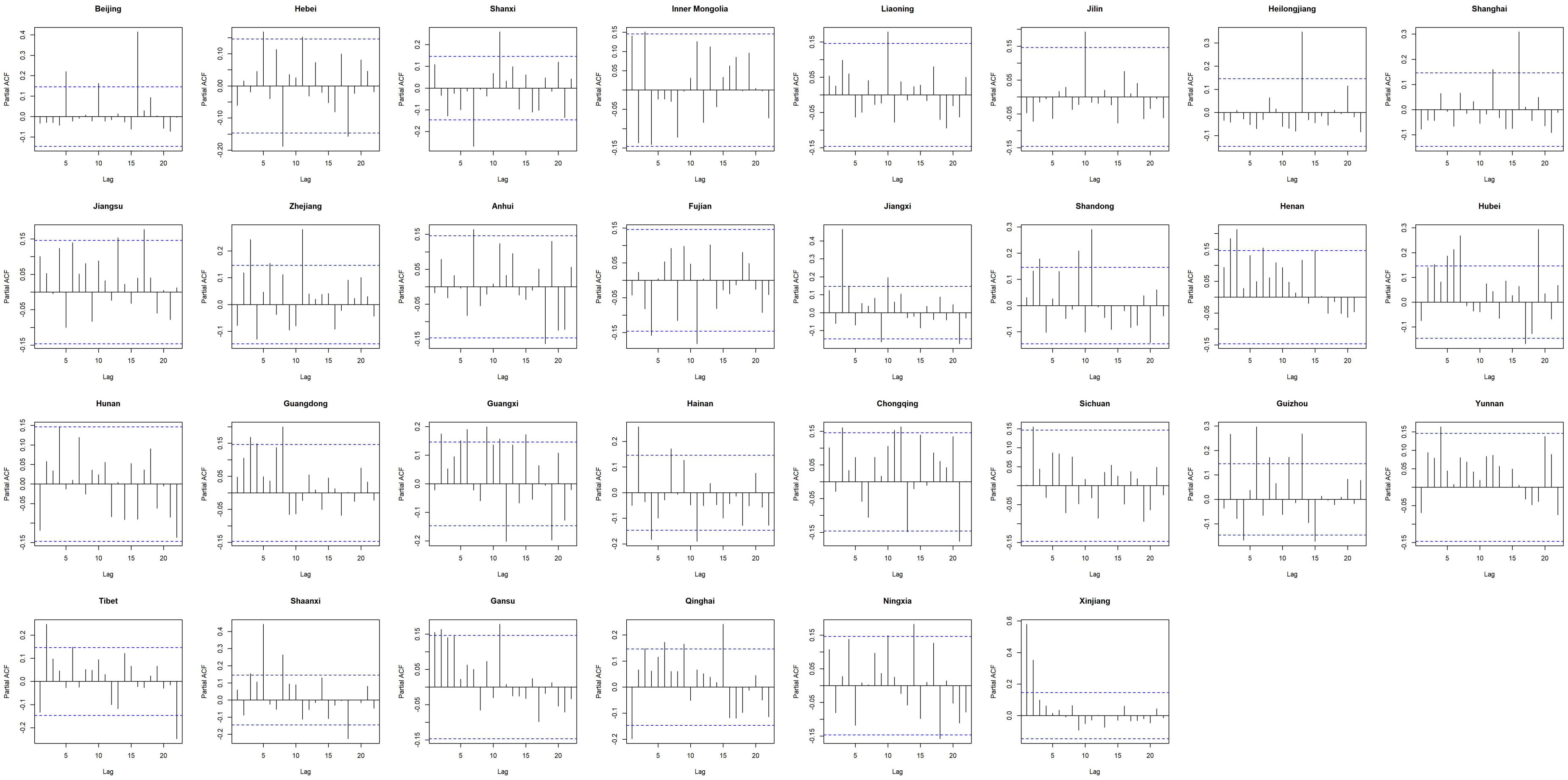

Figure S2. The partial auto-correlation functions of residuals from preliminary analyses for 30 provincial-level administrative divisions with an autoregressive term of residuals.

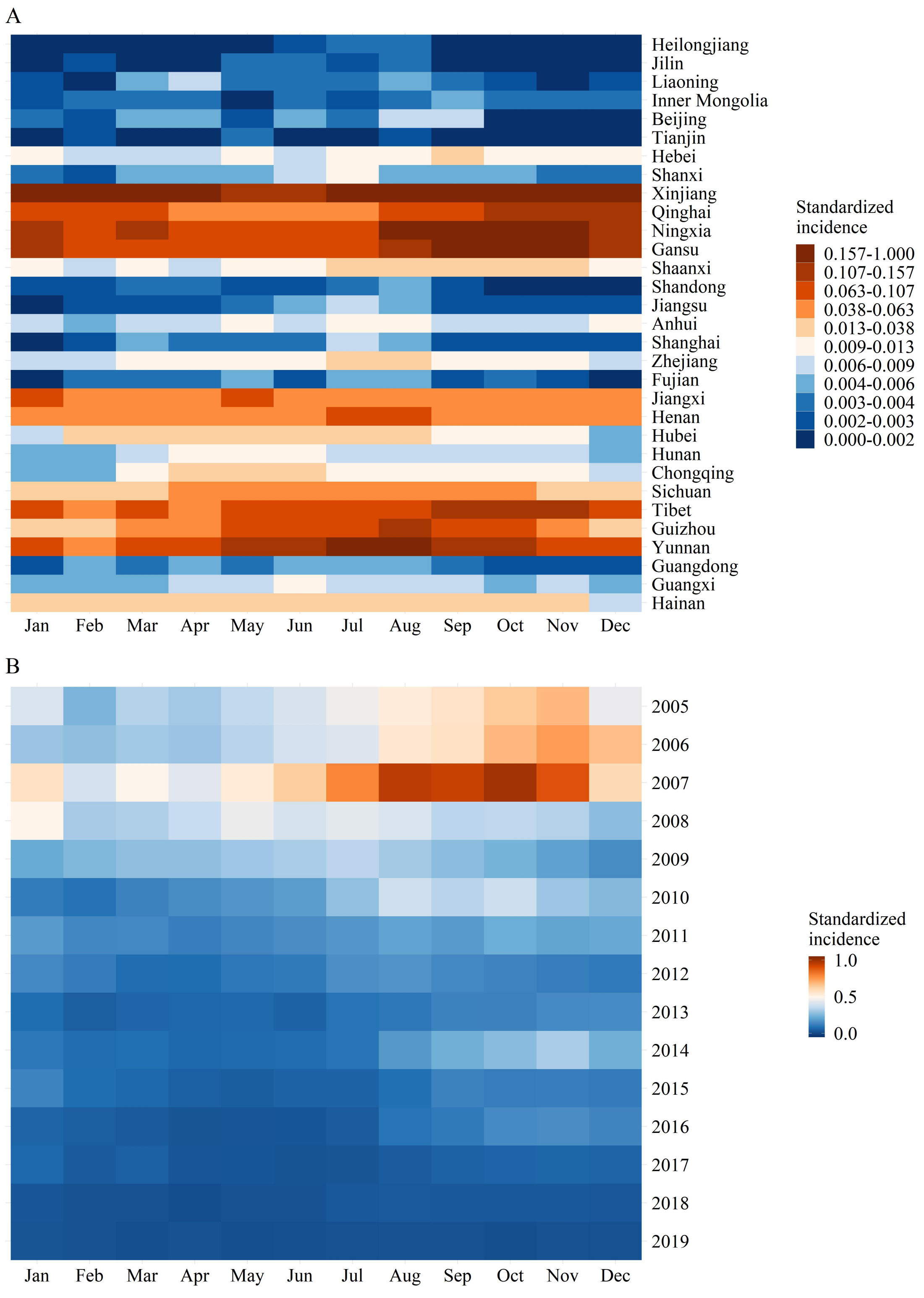

Figure S3. Monthly standardized hepatitis A incidence in the Chinese mainland. The standardized incidence was calculated as the original incidence divided by the maximum incidence during the study period. (A) Monthly standardized hepatitis A incidences of 31 provincial-level administrative divisions; (B) Monthly standardized hepatitis A incidences of different years in the Chinese mainland.

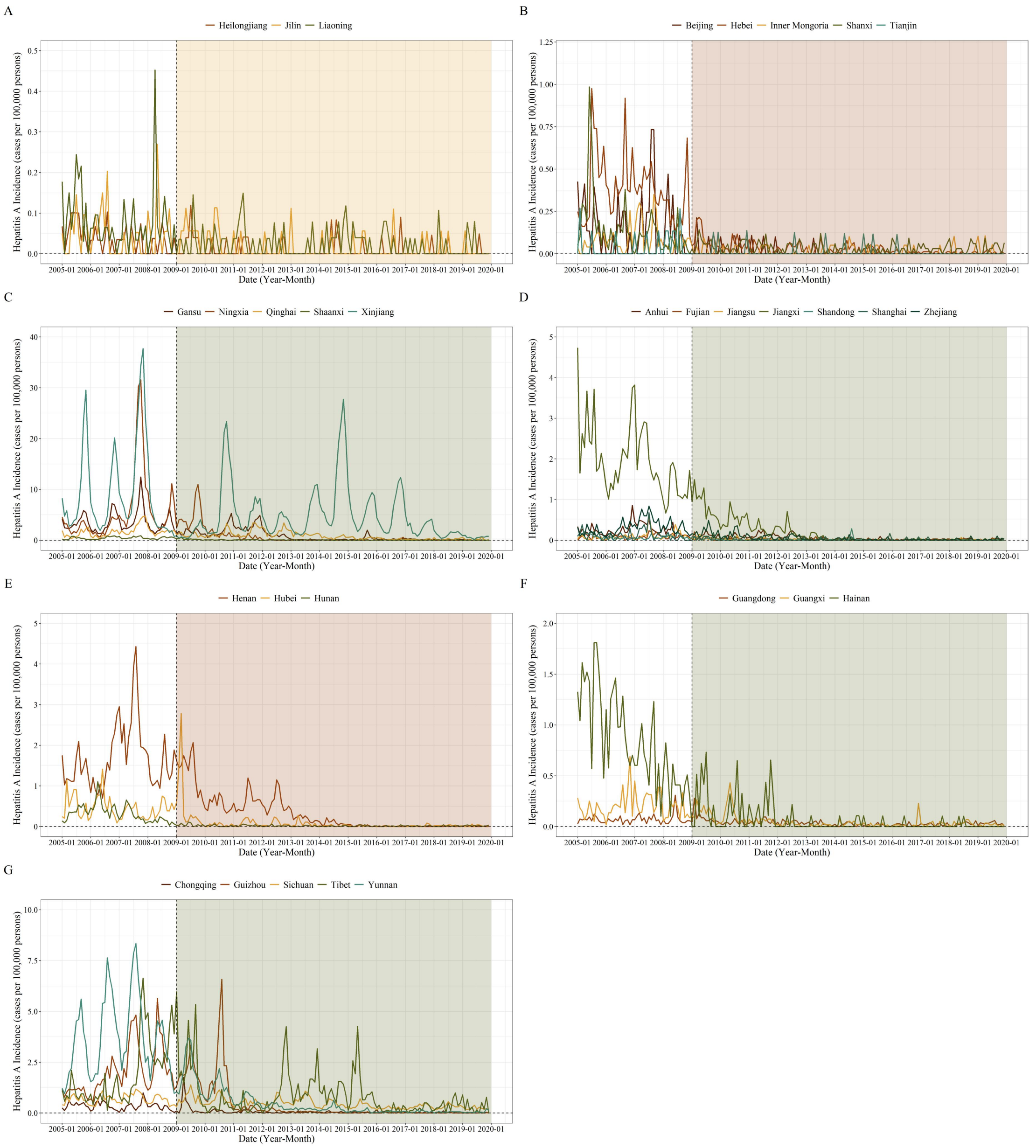

Figure S4. Monthly incidence of hepatitis A among children aged 2-9 years in seven regions of Chinese mainland from 2005 to 2019. The shadow represents the approximate period of the intervention. (A) Northeast China; (B) Northern China; (C) Northwest China; (D) Eastern China; (E) Central China; (F) Southern China; (G) Southwest China.

.

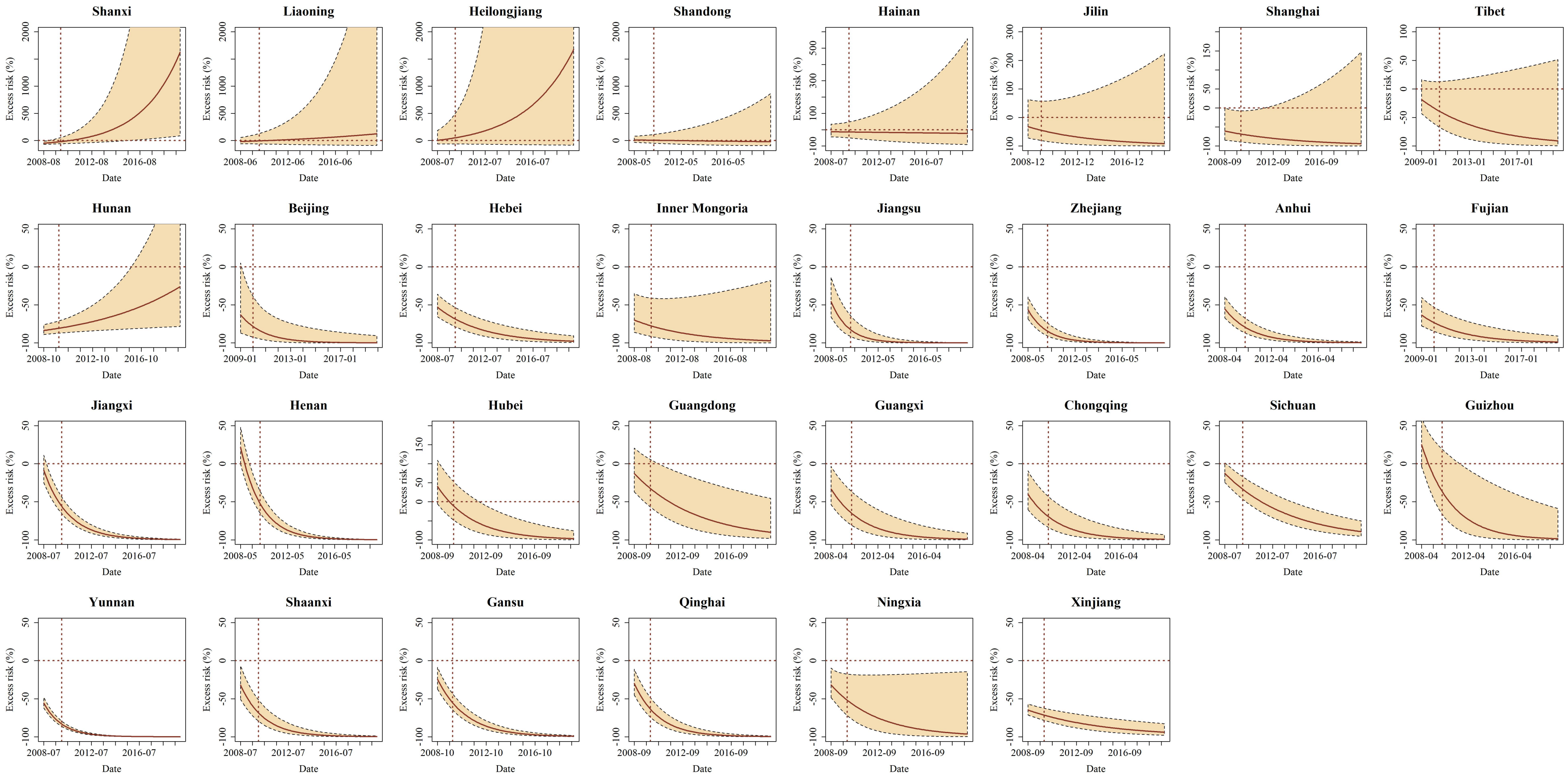

Figure S5. Excess risks of hepatitis A among children aged 2-9 years associated with the Expanded Program on Immunization in 30 provincial-level administrative divisions. The shadow represents the 95% CIs of excess risks.

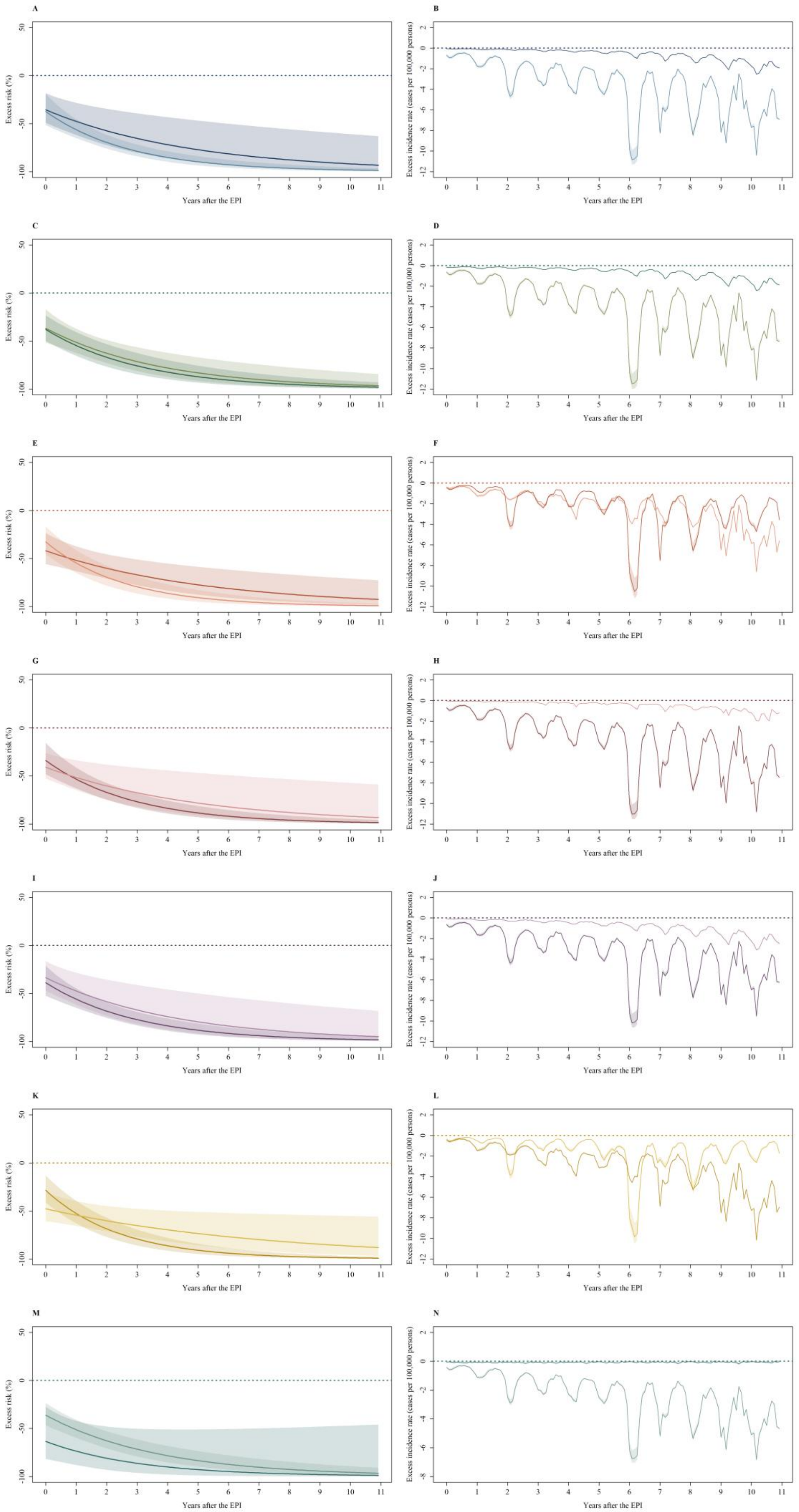
A
B
C
D
E
F
G
H
I
J
K
L
M
N
Excess risk (%)
Excess incidence rate (cases per 100,000 persons)
Years after the EPI

Figure S6. Excess risks and excess incidence rates of hepatitis A among children aged 2-9 years in different subgroups.

In Figure S6 A-L, the dark-colored point estimates and confidence intervals represent the combined effect of PLADs where the value of the variable is greater than or equal to the median and the light-colored point estimates and confidence intervals represent the combined effect of PLADs where the value of the variable is less than the median. In Figure S6 M-N, the dark-colored point estimates and confidence intervals represent the combined effect of PLADs integrating Inactivated vaccines and, the light-colored point estimates and CIs represent the combined effect of PLADs integrating live attenuated vaccines. (A) Urbanization rates; (B) Urbanization rates; (C) GDP per capita; (D) GDP per capita; (E) The number of hospitalization beds per 1,000 persons; (F) The number of hospitalization beds per 1,000 persons; (G) Average incidence of hepatitis A before the implementation of EPI; (H) Average incidence of hepatitis A before the implementation of EPI; (I) The proportion of children; (H) The proportion of children; (K) Illiteracy rates; (L) Illiteracy rates; (M) The type of vaccines; (N) The type of vaccines.

Abbreviation: EPI, Expanded Program on Immunization; PLADs, provincial-level administrative division; GDP, Gross Domestic Product.

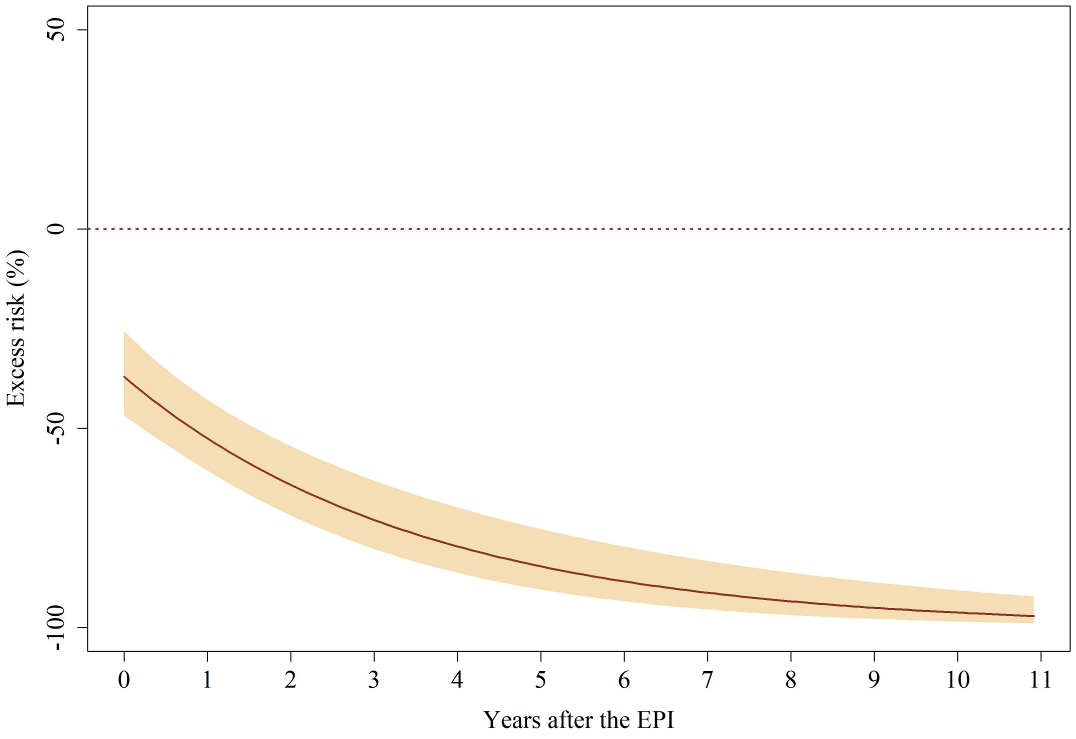

Figure S7. Excess risks of hepatitis A incidence associated with the Expanded Program on Immunization in sensitivity analysis with the replacement of seasonality control. The shadow represents the 95% CIs of excess risks.

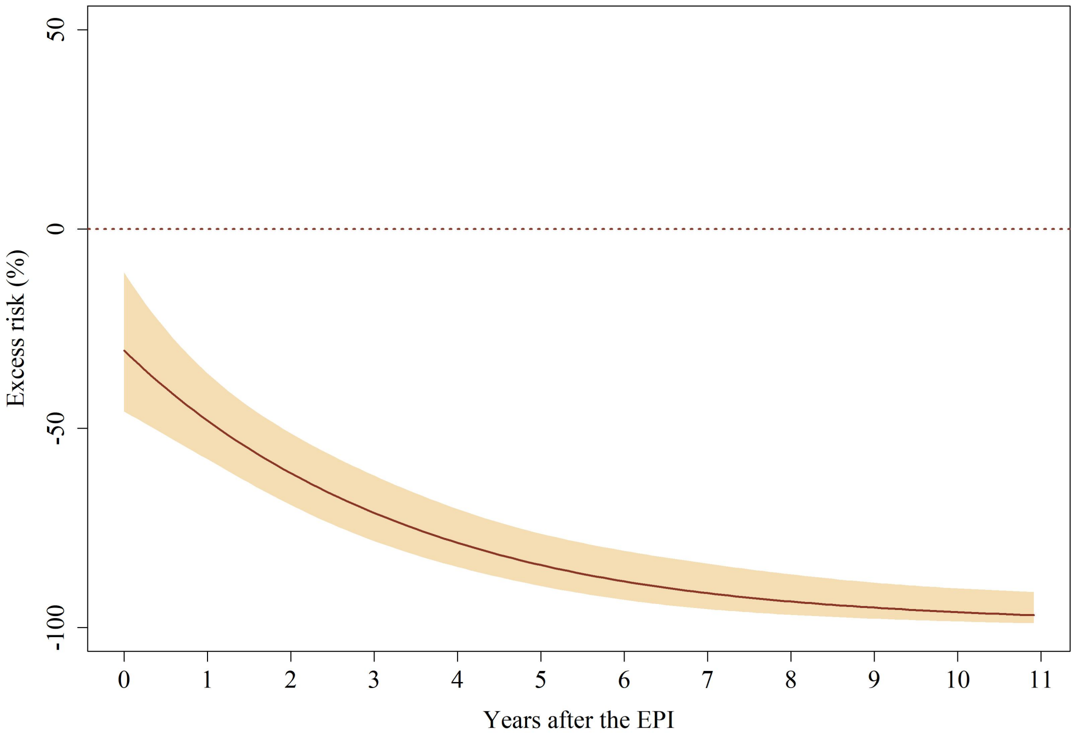

Figure S8. Excess risks of hepatitis A incidence associated with the Expanded Program on Immunization in sensitivity analysis with the non-linear trend. The shadow represents the 95% CIs of excess risks.

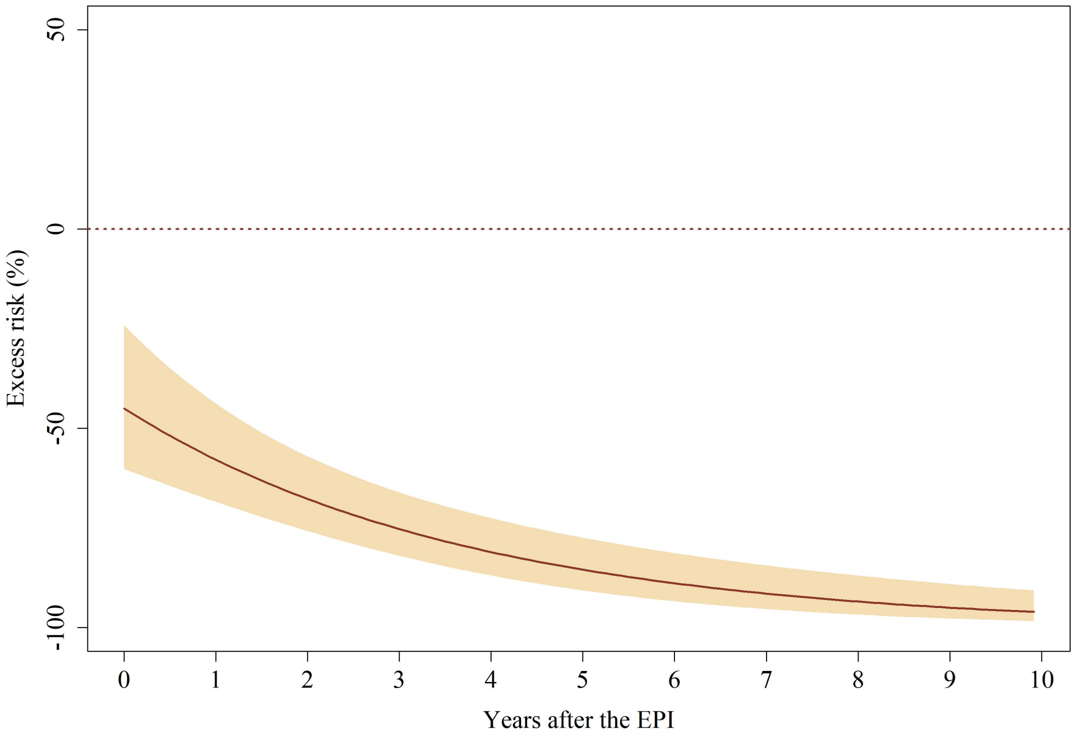

Figure S9. Excess risks of hepatitis A incidence associated with the Expanded Program on Immunization in sensitivity analysis with the transition period. The shadow represents the 95% CIs of excess risks.

Supplemental references

1. Xiao H, Augusto O, Wagenaar BH. Reflection on modern methods: a common error in the segmented regression parameterization of interrupted time-series analyses. Int J Epidemiol. 2021;50(3):1011–5. PMID:33097937
2. Group GFTI. Guideline for the investigation of viral hepatitis A outbreak (version 2021). Chin J Prev Med. 2022;56(5):549–53.
3. Gasparrini A, Leone M. Attributable risk from distributed lag models. BMC Med Res Methodol. 2014;14(1):55. PMID:24758509
4. Lopez BJ, Soumerai S, Gasparrini A. A methodological framework for model selection in interrupted time series studies. J Clin Epidemiol. 2018;103(82–91. PMID:29885427
5. Grijalva CG, Nuorti JP, Arbogast PG, Martin SW, Edwards KM, Griffin MR. Decline in pneumonia admissions after routine childhood immunisation with pneumococcal conjugate vaccine in the USA: a time-series analysis. Lancet. 2007;369(9568):1179–86. PMID:17416262
6. Chinese Ministry of Health. The Implementation of Expanded Programme on Immunization. 2008. http://www.nhc.gov.cn/wjw/ghjh/200804/33518.shtml [20 Mar, 2023].
7. Miu C. Investigation of an outbreak of hepatitis A in primary school students caused by drinking well water. Zhongguo xue xiao wei sheng. 2013;34(12):1517–9.
8. Kong Z. Epidemlology of a hepatitis A outbreak in school. Ji Bing Jian Ce. 2009;24(4):301–2.
9. Cheng H, Yuan H, Xu D, Luo L. Risk factors of an outbreak of hepatitis A in a medical college in Jiangxi province. Xian Dai Yu Fang Yi Xue. 2008;35(2):225–7.
10. Zhu W, Sang C, Miu J. An outbreak of hepatitis A in primary school students. Yu Fang Yi Xue Lun Tan. 2009;15(06):568–9.
11. Song H, He T, Wang Z, Zhao Q, Zhao Z, Shi Y. Investigation and control of an outbreak of hepatitis A in a school. Henan Yu Fang Yi Xue Za Zhi. 2007;18(4):294, 296.
12. Cao Z, Cao Q, Feng J. Investigation on outbreak of hepatitis A in Xixia County. Zhongguo Cheng Xiang Qi Ye Wei Sheng. 2011;26(6):98–9.
13. Rao H, Li P. A town with case-control study of hepatitis a outbreak reasons. Henan Yu Fang Yi Xue Za Zhi. 2014;25(05):353–6.
14. Li X. Investigation and management of a local outbreak of hepatitis A. Hua Nan Yu Fang Yi Xue. 200702):76–7.
15. Peng L, Mei H, Xiong R. Investigation of a waterborne outbreak of hepatitis A in a school. Shi yong yu fang yi xue. 2008;15(3):756–7.
16. He H, He W, Peng Z, Lou C. Investigation of an outbreak of hepatitis A in a middle school. Shi yong yu fang yi xue. 2007;14(6):1782–3.
17. Ma Y, Deng J, Guo N, Huang L, Guo R, Huang C. Epidemiological survey on an outbreak of hepatitis A in a school. Shi yong yu fang yi xue. 2010;17(10):1984–5.
18. Yang X, Lu S, Luo Z. Investigation on outbreak of hepatitis A in a primary school in Yuhong Township, Lingyun County. Ying Yong Yu Fang Yi Xue. 2008;14(2):69.
19. Nong Z, Meng L, Liu Z, Jiang C. Epidemiological investigation of an outbreak of hepatitis A in a school. Zhongguo xue xiao wei sheng. 2009;30(3):274–5.
20. Huang G, Li J. Cohort study on an outbreak of viral hepatitis A in a school of Hechi City in 2010. Yi Xue Dong Wu Fang Zhi. 2013;29(08):859–61.
21. Yang R, Liu W, Lu W, Chen J, Qin Y. Investigation and analysis of an outbreak of viral hepatitis A in Guangxi Zhuang Autonomous Region. Ying Yong Yu Fang Yi Xue. 2017;23(05):389–91.

22. Wang R. Epidemiological investigation of an outbreak of hepatitis A in children left behind. Tai Shan Yi Xue Yuan Xue Bao. 2017;38(9):976–8.
23. Wang B, Xu K, Zhang G. Investigation on An Outbreak of Hepatitis A in Yajiang County in 2007. Yu fang yi xue qing bao za zh. 2008;24(9):701–3.
24. Li C, Zhang P, Chen H, Zhu Y. Investigation on outbreak of hepatitis A in a primary and secondary school in Gao County. Yu fang yi xue qing bao za zh. 2010;26(03):214–6.
25. Du H, Guo Z, Yuan H, Che K. Epidemiological analysis of 3 outbreaks of hepatitis A in township schools. Zhongguo xue xiao wei sheng. 200808):760–1.
26. Che K, Yuan H, Guo Z, Kong X. Epidemiological investigation of an outbreak of hepatitis A in primary school. Xian Dai Yu Fang Yi Xue. 2009;36(7):1362, 1369.
27. Huang Y, Wang D, Yu C, Wu J, Liu Y, Liu B, et al. Epidemiological investigation on the hepatitis A outbreak from contaminated barrels water in a city. Xian Dai Yu Fang Yi Xue. 2009;36(20):3947–8.
28. Huang Y, Wang D, Yu C, Huang M. Investigation of an outbreak of hepatitis A caused by river pollution. Zhongguo Gong Gong Wei Sheng. 2010;26(4):507.
29. Tang G, Zhuang, Zhou J, Wu J, Gong X. Investigation report of hepatitis A outbreak transmission through waterborne. Yi Xue Dong Wu Za Zhi. 2011;27(6):525–7.
30. Ning G, Cheng X, Chen Y, Huang M, Zhuang Y, Huang Y, et al. An epidemiological investigation of hepatitis A outbreak during the period of severe drought in Guizhou Province. Zhongguo Yi Miao He Mian Yi. 2013;19(05):435–8.
31. Lei M, Zhou J, Jiang J, Huang Y, Liu Y. Epidemiology of a hepatitis outbreak epidemiology. Yi Xue Dong Wu Fang Zhi. 2015;31(06):659–60.
32. Zhang R, Wang S, Ji G, Yang X, Tao H. Investigation and analysis of outbreak of hepatitis A in Wuchuan County in 2010. Yu fang yi xue qing bao za zh. 2013;29(12):1047–9.
33. Kong Y, Luo M, Li J. Survey of an hepatitis A outbreak among students after spring outing in Fuming county, Yunnan. Ji Bing Jian Ce. 2009;24(11):896–7.
34. Shi R. Investigation and control of a cluster outbreak of hepatitis A in a pastoral primary school. Yi Xue Xin Xi. 2013;26(10):367.
35. Liu W, Zhao J, Zhao L. Investigation of 191 cases of hepatitis A outbreak. Ningxia yi xue za zhi. 2008;30(10):948–9.
36. Xi J, Wang F, Liu X. Epidemic characteristics of viral hepatitis A in Xiji County, Ningxia from 2006 to 2011. Ningxia yi xue za zhi. 2012;34(07):673–4.
37. Shi J, Yang J. Epidemiological analysis of 52 cases of viral hepatitis A outbreak in a rural kindergarten. Ningxia yi xue za zhi. 2010;32(9):856–7.
38. Li D, Zhang D, Chen C, Tang X, Pu L, Xia M. Investigation of an outbreak of hepatitis A among primary school students in Wenquan County, Xinjiang in 2005. Di Fang Bing Tong Bao. 2007;(02):77.
39. Jiang Z, Li Q, Chen E, Yao J. Epidemic trend of viral hepatitis A in Zhejiang Province. Zhejiang Yu Fang Yi Xue. 2012;24(8):21–3.
40. Li J, Ye Y, Ma Y. Epidemiological analysis on hepatitis A in Henan from 2010 to 2012. Xian Dai Yu Fang Yi Xue. 2014;41(12):2260–1, 2265.
41. Li J, Yang K, Du B, Zhang X, Shi L, Ji Y, et al. Epidemiological characteristics of hepatitis A in Henan province,1990-2020. Zhongguo Yi Miao He Mian Yi. 2021;27(6):659–62, 672.
42. Li L, Li N, Hu Y, Liu N, Yu C, Wang L, et al. Analysis and prediction of the epidemiological

characteristics of hepatitis A in Hubei Province from 2004 to 2019. Zhonghua ji bing kong zhi za zhi. 2020;24(10):1165–9.

43. Zhang R, Tao H, Ji G, Wang S, Wu M, Chen W. Analysis of epidemiological characteristics of hepatitis A in Zunyi City from 2001 to 2012. Xian Dai Yu Fang Yi Xue. 2014;41(21):3855–6, 3871.
44. Kong Y, Ding Z, Pang Y, Luo M, Tao R, Kang W. Analysis of epidemiological characteristics of viral hepatitis A in Yunnan Province from 2004 to 2008. Xian Dai Yu Fang Yi Xue. 2011;38(20):4261–2.
